# Traditional Chinese medicine residues promote the growth and quality of *Salvia miltiorrhiza* Bunge by improving soil health under continuous monoculture

**DOI:** 10.3389/fpls.2023.1112382

**Published:** 2023-06-07

**Authors:** Sha Liu, Guang Yang, Faming Wu, Yang Ge, Fusong Liu, Chunjuan Pu, Zihan Wang, Ye Shen, Xiuteng Zhou, Yuzhi Luo, Fengsheng Li, You Zhang, Meilan Chen, Luqi Huang

**Affiliations:** ^1^ School of Pharmacy, Chengdu University of Traditional Chinese Medicine, Chengdu, Sichuan, China; ^2^ State Key Laboratory of Dao-di Herbs, National Resource Center for Chinese Materia Medica, China Academy of Chinese Medical Sciences, Beijing, China; ^3^ School of Pharmacy, Zunyi Medical University, Zunyi, Guizhou, China; ^4^ Cultivation Base Department, Laiwu Purple Light Ecological Park Co., Ltd., Jinan, Shandong, China

**Keywords:** *S. miltiorrhiza*, Chinese medicinal herbal residue, soil physicochemical properties, soil microbial community, continuous cropping

## Abstract

Continuous monoculture of crops has resulted in reduced yields and quality, as well as soil deterioration. Although traditional Chinese medicine residues (TCMRs) are known to promote plant growth and soil health, few studies have investigated their effectiveness in continuous monoculture soils. Here, we studied the impact of chemical fertilizers (CF) and four TCMRs with antibacterial activities on the growth of *S. miltiorrhiza* (a widely used medicinal plant in China), accumulation of active ingredients in plants, and soil health under continuous monoculture conditions. Compared with no fertilizer (CK) and CF, fermented *Sophora flavescens* radix residue (SFRf) and fermented and unfermented *Moutan cortex* residue (MCRf and MCRu, respectively) resulted in a reduction of the disease index of root rot, while CF did not. The CF and four TCMR treatments increased the accumulation of nitrogen (N) (42.8-124.6% and 17.0-101.7%), phosphorous (P) (19.8-74.7% and 8.3-27.4%), and potassium (K) (104.1-212.0% and 9.3-51.8%) in shoots and roots compared to CK. The differences in nutrient accumulation between the CF and TCMR treatments were statistically insignificant, excepted for the N accumulation in the roots. All fertilization treatments increased plant biomass compared to CK, with increases of 25.57-89.86% and 2.62-35.28% in shoots and roots, respectively. The SFRf treatment exhibited the most significant enhancement in both shoot and root biomass. CF significantly reduced the accumulation of seven active ingredients in roots by 23.90-78.95% compared to CK, whereas each TCMR increased accumulation of certain active ingredients. The TCMR treatments effectively improved the health of deteriorated soil by enhancing soil physicochemical properties, restoring the balance of the microbial community, recruiting beneficial bacteria, and reducing the relative abundance of the pathogen *Fusarium.* The SFRf treatment exhibited superior performance in improving soil health than other treatments. Overall, the TCMRs outperformed CF in restoring soil health and promoting the yield and quality of *S. miltiorrhiza*. These findings offer guidance for improving the health of continuous cropping soil and recycling TCMRs.

## 1 Introduction

*S. miltiorrhiza* is a widely used medicinal plant in China for treating cardiovascular diseases (Li et al., 2020), with annual global consumption exceeding 20 million kilograms (Pang et al., 2016). Due to the limited availability of wild resources, artificially cultivated *S. miltiorrhiza* is primarily used for clinical purposes and producing Chinese patent medicine. The limited arable land suitable for growing *S. miltiorrhiza*, caused by geographical and climatic factors, leads to continuous monoculture in the main production area. Long-term monoculture of crops inhibits plant growth, aggravates diseases, and ultimately reduces quality and yields. These obstacles posed by continuous monoculture severely impede the sustainable development of crop production (Chen et al., 2022b). Continuous monoculture caused substantial reductions in the fresh root biomass, taproot diameter, and total chemical composition of *S. miltiorrhiza* (Liu et al., 2020; Wang, 2020).

Soil health is defined as soil’s continuous ability to function as a vital living ecosystem that supports plants, animals, and humans (Nunes et al., 2020). This definition emphasizes that soil health is a dynamic, life-sustaining condition that supports the macro- and microorganisms, nutrient cycling, and physical properties needed to provide food, feed, fiber, fuel, and shelter for sustaining life and protecting water and air quality (Nunes et al., 2020). Soil degradation is a significant contributor to the obstacles related to continuous monoculture. Continuous monoculture leads to poor soil physicochemical properties, including decrease in nutrients (Yu et al., 2017; Li et al., 2019). Moreover, studies have shown that continuous monoculture disrupts the structure of the soil microbial community, resulting in a decrease in beneficial functional microbes and an increase in harmful pathogenic fungi within plant rhizosphere soil (Wang et al., 2018a; Chen et al., 2022b). The soil microbial community, including its composition, diversity, and community stability, has become a crucial indicator for assessing soil health (Hermans et al., 2017; Lehmann et al., 2020). Continuous monoculture of *S. miltiorrhiza* led to decreased contents of total phosphorus, total potassium, available phosphorus, and other nutrients in soils (Wang et al., 2019b). A decrease in bacterial community diversity and an increase in pathogenic fungal community diversity were found in the continuous monoculture soil of *S. miltiorrhiza* (Liu et al., 2019; Zhou et al., 2019). Furthermore, the relative abundance of *Fusarium*, the primary pathogen causing root rot of *S. miltiorrhiza* (Wang et al., 2018b; Pu et al., 2022), increased in continuous monoculture soils (Wang et al., 2019b).

Plant residues can have various positive effects on soil, such as increasing the organic matter content, improving physical properties, supplying nutrients, and enhancing beneficial microbial diversity and activity (Tejada et al., 2009; Chen et al., 2016c; Dignam et al., 2019). However, distinct types of plant residues may exhibit varying impacts on soil physicochemical properties and microbial communities due to their unique compositions (Chang et al., 2022; Chen et al., 2022a). TCMRs are plant residues left over after extracting active ingredients from medicinal plants. They are rich in nutrients, such as organic matter, trace elements, and other essential nutrients (Ma et al., 2004; Jia et al., 2010). Additionally, they have a light texture and good air permeability (Chen et al., 2016a). These characteristics make TCMR an environmentally friendly and economical resource for soil improvement in agricultural production. Applicating TCMRs in crop production effectively enhances plant growth by increasing organic carbon and inorganic nutrient levels, stimulating microbial activity, and increasing soil enzyme activity (Niu et al., 2013; Chen et al., 2016b; Ma et al., 2019b). However, extensive trials of TCMRs as soil amendment are lacking. The annual production of TCMRs in China is 60-70 million tons (Lu and Li, 2021). Unfortunately, most are incinerated, stacked, or landfilled as waste (Meng et al., 2018). Besides wasting valuable TCMR resources, these methods cause severe environmental pollution (of soil, water, and air) (Lu and Li, 2021).

*Sophora flavescens* radix (SF) and *Moutan cortex* (MC) are traditional Chinese medicines (TCMs) with antimicrobial and insecticidal properties. In agriculture, extracts of SF and MC serve as biopesticides (Xia and Chen, 1999; Fang et al., 2018). The chemical composition of SF contains alkaloids, such as matrine and oxymatrine. Matrine applied to continuous cropping soils significantly reduced the abundance of *F. solani*, *F. proliferatum*, *F. moniliforme*, and *F. oxysporum* compared to the control (Wang et al., 2019a). The chemical composition of MC contains paeonol and paeoniflorin. A study demonstrated that paeonol effectively inhibited various plant pathogens, including *Valsamali*, *F. oxysporum*, and *Pseudomonas solanacearum* (Yang et al., 2021). We speculate that applying *Sophora flavescens* radix residue (SFR) and *Moutan cortex* residue (MCR) into the continuous monoculture soil can enhance soil health by providing nutrients as plant residues and directly inhibiting pathogen growth through their active ingredients. However, no study has examined the effects of SFR and MCR on the health of deteriorated continuous monoculture soils. In this study, four types of TCMR, including fermented and unfermented SFR and fermented and unfermented MCR, were added to soils used for continuous monoculture *S. miltiorrhiza*. We hypothesize that applying TCMRs will (1) alleviate diseases of *S. miltiorrhiza* caused by continuous monoculture, (2) promote the growth of continuous monoculture *S. miltiorrhiza* and accumulation of active ingredients, (3) and improve soil physicochemical properties and restore the balance of microbial community structure in continuous monoculture soil. The results will offer guidance on enhancing the health of continuous cropping soil and reusing Chinese medicine residues.

## 2 Materials and methods

### 2.1 Experimental materials and experimental design

The experiment was performed in Miaoshan Town, Laiwu District, Jinan City, Shandong Province, China (36°13′37′′N, 117°41′15′′E) from March to October 2021, which is a long-term experimental base for *S. miltiorrhiza* cultivation used by the China Academy of Chinese Medical Sciences. This location falls within the temperate monsoon climate zone of North China, characterized by a warm continental monsoon climate. The annual average precipitation, temperature, and relative humidity are 490.5-695.3 mm, 8.5-18.8°C, and 62%, respectively. From July to September, almost 70% of the total precipitation occurs.

The experiment was conducted in pots with a bottom diameter of 14.4 cm, top diameter of 19.5 cm, and height of 19.4 cm. The substrate used for the pot experiment was field soil that had undergone continuous cultivation with *S. miltiorrhiza* for one year, classified as sandy loam. **Table 1** shows the soil physicochemical properties. The chemical fertilizer used in this study was identical to that employed in actual production and obtained from Shandong Zhongtianhua International Chemical Fertilizer Import and Export Co., Ltd. (Shandong, China). The fermented SFR and MCR were obtained by natural composting in an open outdoor area without any added substances. The windrow was triangular, with a bottom width of 1.5 m and a height of 1.2 m, and turned the windrow when its temperature exceeded 65°C (Tirado and Michel, 2010; Sun et al., 2014). The fermentation period was approximately three months. The SFR and MCR had to be completely decomposed, black-brown, and odorless. And the germination rate of *S. miltiorrhiza* seeds treated with residue extract needed to exceed 70%. Then, the residues were air-dried and pulverized. **Table S1** shows the nutrient composition of the TCMRs.

**Table 1:** Soil physicochemical properties and *Fusarium* abundance before planting.

Treatment	pH	OM%	CEC cmol/kg	TN mg/kg	TP mg/kg	TK mg/kg	AN mg/kg	AP mg/kg	AK mg/kg	*Fusarium* abundance
**CK**	6.38	1.03	8.49	565.50	358.02	8763.69	13.97	18.54	54.31	29.51%
**SFRf**	6.47	1.21	9.45	663.57	384.73	8870.11	9.99	16.62	57.01	9.01%
**SFRu**	6.42	1.15	9.91	682.40	359.19	8615.93	9.40	16.59	55.08	6.43%
**MCRf**	6.43	1.91	10.22	1017.82	409.97	8778.30	11.84	22.07	61.33	12.03%
**MCRu**	6.52	1.83	10.20	789.02	368.79	8151.86	6.16	15.50	63.67	8.58%

OM, organic matter; CEC, cation exchange capacity; TN, total nitrogen; TP, total phosphorus; TK, total potassium; AN, available nitrogen; AP, available phosphorus; AK, available potassium.

The experimental treatments were as follows: (1) CK: no fertilizer; (2) CF: chemical fertilizer; (3) SFRf: fermented SFR; (4) SFRu: unfermented SFR; (5) MCRf: fermented MCR; and (6) MCRu: unfermented MCR. CF, a combined fertilizer with 18% N, 6% P, and 10% K, was applied at the flowering stage of *S. miltiorrhiza* in the form of topdressing according to the fertilization measure adopted in actual production. The amount of CF applied per pot was calculated based on the commonly used concentration of 1200 kg·ha^-1^ in the field and a plant density of 6.67×10^4^ plants·ha^-1^. TCMR powder was added to soils at 0.5% by soil weight 40 days before planting (Ma et al., 2019b). Seedlings were grown in the field using seeds with a germination rate of approximately 75%. On May 8, 2021, two three-month-old healthy *S. miltiorrhiza* seedlings with uniform growth, about 10 cm in length, were transplanted into individual pot. Twenty pots were allocated to each treatment and the potted plants were situated in an open field, with all treatments receiving identical management measures. Potted plants were watered once or twice a week, depending on the weather, thoroughly but without spilling out of the pot (0.5~0.8 L per pot each time). The pots from the same treatment were placed together. The pots from different treatments were arranged at random.

### 2.2 Sample collection

During the harvest period in October 2021, the rhizosphere soils (the soil layer< 2 mm thick surrounding the root) of *S. miltiorrhiza* were collected and passed through a 2 mm sieve. The soil sample from each treatment was divided into two portions: the first was transferred into sterile tubes and stored at -80°C until microbial community analysis; the second was air-dried to determine the physicochemical properties. All sampling equipment for microbial analysis was sanitized with 75% alcohol.

After collecting the rhizosphere soil, the remaining roots were collected to analyze the root microbial community. Because the different growth and development zones of plant roots have various microbial community structures (Yu et al., 2021), we selected the same section of branch roots (the 4-5 cm section in the middle of the roots) with roughly equal diameters (3-5 mm) for microbial community analysis to avoid differences caused by different space zones and excessive differences of root diameter. Roots were transferred into sterile tubes, washed five times (each time for 30 s, in a new tube) with sterile phosphate buffer solution (PBS) (Beijing Solarbio Science and Technology Co., Ltd.) using ultrasonication, transferred into new sterile tubes, and stored at -80°C until microbial community analysis.

After the abovementioned sampling procedure, the remaining *S. miltiorrhiza* plants were collected and naturally dried in the shade to determine the biomass and the accumulation of nitrogen, phosphorous, potassium, and active ingredients.

### 2.3 Disease index of root rot

The incidence and severity of plant diseases were recorded during the *S. miltiorrhiza* harvest period, and a disease index was calculated. The grading criteria for root rot was as follows: in grade 0, no disease; in grade 1, the root lesion area is less than 25%; in grade 2, it ranges from 25% to 50%; in grade 3, it ranges from 50% to 75%; and in grade 4, the root lesion area exceeds more than 75%, or the plant has died.(Wu et al., 2021)


$$Diseaseindex=\frac{\sum \left(\text{number of diseased plants at each grade}\times \text{ disease grade value}\right)\;}{\text{total number of investigated plants}\times \text{maximum disease grade}}\times 100$$

### 2.4 Assessment of plant biomass

The weights of the shoots and roots from each plant were recorded after drying. The tissues were subsequently ground, passed through a 60-mesh sieve, and refrigerated at 4°C until determined the contents of nitrogen, phosphorous, potassium, and active ingredients in the tissues.

### 2.5 Determination of active ingredients in plant tissues

The shoots and roots were analyzed for the contents of three water-soluble ingredients (caffeic acid, rosmarinic acid, and salvianolic acid B) and four liposoluble ingredients (dihydrotanshinone I, cryptotanshinone, tanshinone I, and tanshinone IIA). The certified reference materials were purchased from Sichuan Weiqi Biological Technology Co., Ltd. (China). The active ingredients were extracted via ultrasonic extraction using 75% methanol as the solvent (Yang et al., 2019). An HPLC analysis method, optimized based on the approach described by Pu et al. (2021), was employed to determine the active ingredient contents.

### 2.6 Soil physicochemical properties and nutrients in plant tissues

The soil pH was determined using a pH meter (Mettler Toledo, Switzerland) in a 1:5 soil/water suspension (Bao, 2000). The content of organic matter (OM) was determined by a Vario Macro CNS Analyzer (Elementar, Germany) after the catalytic combustion of finely ground samples (Bao, 2000). The cation exchange capacity (CEC) of soils was determined with the EDTA-ammonium acetate exchange method (Bao, 2000). The available nitrogen (AN) content was detected using the alkaline hydrolysis-diffusion method (Bao, 2000). The available phosphorus (AP) was extracted with NaHCO_3_ and detected using the method of Olsen et al. (1954). The available potassium (AK) was extracted with NH_4_OAc and detected using the flame photometric (Bao, 2000). For total nitrogen (TN) analysis, soils and plant tissue powders were digested with H_2_SO_4_ (Wolf, 2008), and the TN content was detected using a K1100F nitrogen analyzer (Haineng, China). For total phosphorous (TP) and total potassium (TK) analysis, plant tissues were digested with H_2_SO_4_ and H_2_O_2_ (Wolf, 2008), soils were digested with HNO_3_, HCI, and HClO_4_ (Bao, 2000), and the TP and TK contents were detected using an AI1200 atomic absorption spectrometer (Aurora, Canada).

### 2.7 Microbial community measurements

DNA extraction, PCR amplification, and amplicon sequencing were conducted at Beijing Novogene Co., Ltd. DNA of the root samples were extracted using the CTAB method (Lutz et al., 2011), and DNA of the soil samples were extracted using a Magnetic Soil and Stool DNA Kit (Qiagen, Valencia, California, USA) according to the manufacturer’s protocol. The V5-V7 region of the 16S rRNA gene of bacteria was amplified using specific primers 799F: AACMGGATTAGATACCCKG, 1193R: ACGTCATCCCCACCTTCC (Bao et al., 2021), and the ITS1 region of the fungi was amplified using specific primers ITS5-1737F: GGAAGTAAAAGTCGTAACAAGG, ITS2-2043R: GCTGCGTTCTTCATCGATGC (Liu et al., 2019). The purified PCR products were sequenced using the Illumina NovaSeq6000 platform (Illumina, USA).

### 2.9 Comprehensive evaluation of the effects of TCMRs on soil health and the growth and quality of *S. miltiorrhiza*

The Z score (Rahman et al., 2009; Wulanningtyas et al., 2021) was used to comprehensively evaluate the effects of different treatments on soil health and the yield and quality of *S. miltiorrhiza*. The indexes for soil health included the soil physicochemical properties (pH, CEC, OM content, and C/N ratio), bacterial diversity and fungal diversity in rhizosphere soils (observed species, Shannon, Simpson, Chao 1, and ACE indexes), and the relative abundance of *Fusarium*. Due to the rapid release of nutrients from CF, the total N, P, and K and available N, P, and K levels in the soils increase rapidly in the short term. In the long run, an evaluation of soil fertility based on total N, P, and K and available N, P, and K levels lacks an objective. Hence, these indexes were excluded from the comprehensive assessment. The indexes for plant growth and quality included the yield of *S. miltiorrhiza* and the accumulation of seven active ingredients. The Z score for a particular variable in a specific treatment was computed using the following formula:


$${\text{Z}}_{\text{i}}=\frac{{\text{X}}_{\text{i}}-\bar{\text{X}}}{\text{S}}$$

Zi: standardized score; Xi: mean value for a particular variable in a specific treatment; $\bar{X}$ : the mean value of all treatments; S: the population standard deviation of the mean value.

The total score for each treatment was calculated by summing the Z scores of all variables. The treatment with the highest total score was deemed optimal for improving the health of continuous monoculture soils and promoting the growth and quality of *S. miltiorrhiza*.

### 2.9 Statistical analysis

The soil physicochemical properties, biomass, and accumulation of nutrients and active ingredients in *S. miltiorrhiza* among different treatments were statistically analyzed using SPSS Statistics (version 26, IBM, USA). The significance of differences was evaluated using a one-way analysis of variance (ANOVA) and Tukey’s test. Statistical significance was determined at P< 0.05 for differences observed among treatments. GraphPad Prism (version 8.0, California) and Adobe Illustrator 2020 (Adobe Systems Inc., USA) were used to prepare the figures.

Raw data from the microbial sequencing were merged and filtered, then removed the chimeric sequences to obtain effective tags. Next, sequences with ≥97% similarity were assigned to the same OTU by UPARSE software (Edgar, 2013) (version 7.0.1001). The taxonomic information for 16S and ITS OTUs was annotated using the SILVA database (Quast et al., 2012) and UNITE database (Kõljalg et al., 2013), respectively. The alpha diversity indexes of the microbial communities were calculated with QIIME (Version 1.7.0) and displayed with R software (Version 2.15.3). Principal Coordinate Analysis (PCoA) was conducted to investigate the dissimilarity in community composition and visualized using ade4 and ggplot2 packages in R software (Version 2.15.3). Unweighted pair-group method with arithmetic mean (UPGMA) clustering was performed to analyze the similarity of different samples using QIIME software (Version 1.9.1). The enriched species in various treatments were analyzed using LEfSe software. Graphviz-2.38.0 was used to draw the network diagram. Because the microbial analysis results for one root sample in the MCRu treatment were abnormal, five replicates were used in all the microbial community analyses to ensure a consistent sample number.

## 3 Results

### 3.1 TCMRs alleviate root rot and promote the growth and quality of *Salvia miltiorrhiza* under continuous monoculture

The CK treatment exhibited the highest incidence rate (67.5%) and disease index (23.75) of root rot (**Figure S1**). TCMRs mitigated the damage caused by root rot in *S. miltiorrhiza*, while CF did not. The SFRf, MCRf, and MCRu treatments significantly reduced the incidence rate and disease index compared to CK, with decreases of 48.15-59.26% and 44.74-73.68%, respectively.

**Figure 1:**
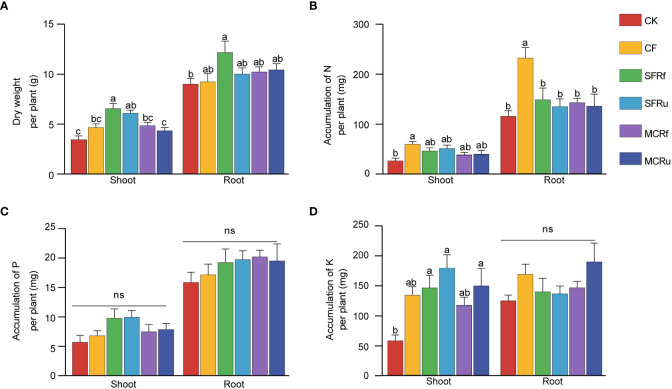
Effect of TCMRs on the growth of *S. miltiorrhiza* **(A)** The biomasses of different tissues of *S. miltiorrhiza* in different treatments (n=13); **(B–D)**: The accumulation of N, P, and K per *S. miltiorrhiza* plant (n=6). Different letters indicate significant differences between treatments at P< 0.05. ‘ns’ indicates that the difference among different treatments is not significant. The error bar above each column represents the SEM value. CK, no fertilizer; CF, chemical fertilizer; SFRf, fermented *Sophora flavescens* radix residue; SFRu, unfermented *Sophora flavescens* radix residue; MCRf, fermented *Moutan cortex* residue; MCRu, unfermented *Moutan cortex* residue.

Both shoot and root biomass of *S. miltiorrhiza* were highest in the SFRf treatment, 89.86% and 35.28% higher than that in the CK treatment, respectively (*P*<0.05) (**Figure 1A**). Compared with CK, the shoot biomass in the SFRu treatment significantly increased by 76.48%, and the root biomass of the SFRu treatment exhibited an increasing trend with no significant difference. Additionally, compared with the CF treatment, the shoot biomass of the SFRf treatment increased significantly by 41.05%. The CK treatment exhibited the highest root-shoot ratio despite having the least amount of root biomass, which was significantly greater than that observed in the SFRf and SFRu treatments (**Figure S2**). It suggested that the four TCMR treatments promoted the growth of plants, particularly the shoots. Based on these findings, the application of SFRf demonstrated the best efficacy in promoting the growth of *S. miltiorrhiza*.

The accumulation of N in both shoots and roots significantly increased in the CF treatment compared to CK, with increases of 124.6% and 101.7%, respectively (**Figure 1B**). Both the CF and TCMR treatments had no significant effects on the accumulation of P in plants; however, the TCMR treatments tended to increase P accumulation more than CF (**Figure 1C**). Compared with CK, the accumulation of K in shoots significantly increased in the SFRf (154.8%), SFRu (212.0%), and MCRu (160.4%) treatments (**Figure 1D**).

**Figure 2:**
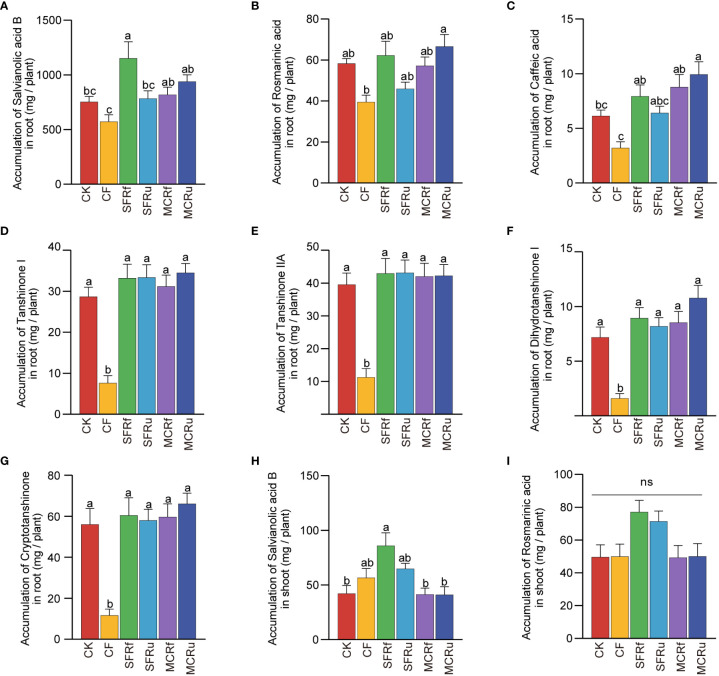
Effect of TCMRs on the accumulation of active ingredients in *S. miltiorrhiza* **(A–G)**: The accumulation of salvianolic acid B, rosmarinic acid, caffeic acid, tanshinone I, tanshinone IIA, dihydrotanshinone I, and cryptotanshinone in roots. **(H, I)**: The accumulation of salvianolic acid B and rosmarinic acid in shoots. The error bar above each column represents the SEM value. Different letters indicate significant differences between treatments at P< 0.05 (n=13). ‘ns’ indicates that the difference among different treatments is not significant.

The lowest accumulation of each active ingredient in roots was observed in the CF treatment (**Figures 2A–G**), with values 23.90-78.95% lower than those in the CK treatment. The SFRf treatment significantly increased salvianolic acid B accumulation in roots by 52.95% compared with CK (**Figure 2A**). The MCRu treatment significantly increased the accumulation of caffeic acid in roots by 61.91% compared to CK (**Figure 2C**). There was no significant difference in the accumulation of active ingredients in shoots between the CK and CF treatments (**Figures 2H, I**, **Figure S3**). However, the application of SFRf significantly increased the accumulation of salvianolic acid B in shoots compared to CK (**Figure 2H**). The results showed that the application of CF significantly reduced the accumulation of all active ingredients in roots; the four TCMRs exhibited varying degrees of efficacy in promoting the accumulation of specific ingredient, with SFRf demonstrating the best effect.

### 3.2 TCMRs improve soil physicochemical properties

**Figure 3:**
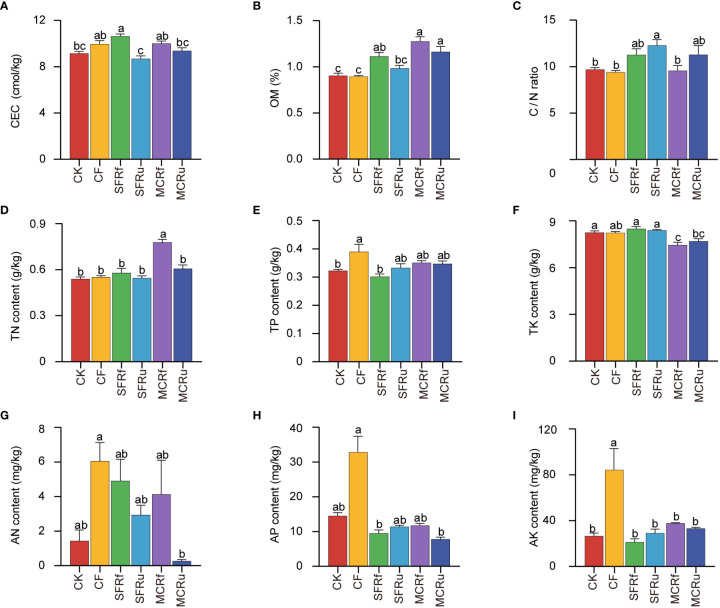
Effect of TCMRs on the soil physicochemical properties **(A)**: cation exchange capacity (CEC) of soil; **(B)**: organic matter (OM) content; **(C)**:ratio of C content to N content (C/N). **(D)** total nitrogen (TN) content; **(E)** total phosphorus (TP) content; **(F)** total potassium (TK) content; **(G)** available nitrogen (AN) content; **(H)** available phosphorus (AP) content; **(I)** available potassium (AK) content. The error bar above each column represents the SEM value. Different letters indicate significant differences between treatments at P < 0.05. (n=6).

Compared with CK, the application of TCMRs (except for SFRu) significantly increased the OM content by 23.02-40.88%, and the application of CF significantly increased the contents of TP and AK (**Figure 3**). In addition, the SFRf treatment significantly increased CEC by 16.30% compared to CK. The SFRu treatment significantly increased the C/N ratio by 30.56%. The soil acidification caused by CF was more pronounced than that of the TCMR treatments (**Figure S4**). These results indicated that the application of SFRf performed best in increasing soil OM and CEC, and CF performed best in increasing soil total P and available K.

### 3.3 TCMRs improve the microbial community structure of the soils and roots of *Salvia miltiorrhiza*

#### 3.3.1 Effect of TCMRs on the diversity and composition of the microbial community of rhizosphere soils and roots of *Salvia miltiorrhiza*

**Figure 4:**
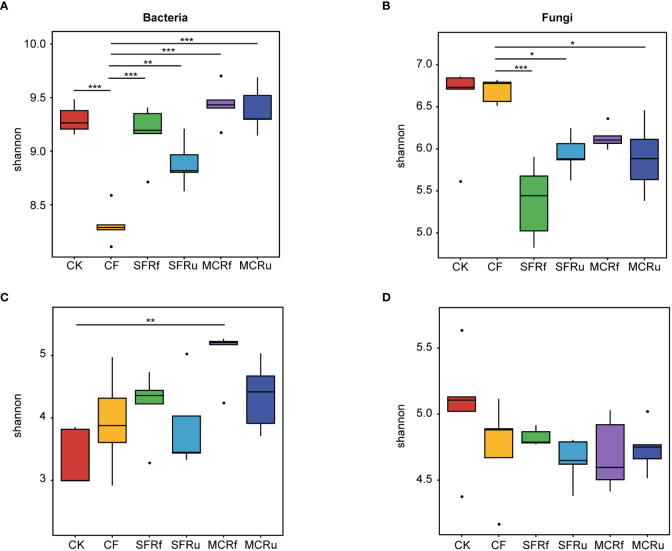
Effects of TCMRs on the alpha diversity of the microbial communities in the rhizosphere soils and roots of *S. miltiorrhiza* at harvest The Shannon index indicates the microbial diversity in each treatment. The box chart shows the median, maximum, minimum, and abnormal values within the group. The higher the Shannon index is, the higher the diversity of the community. The Tukey's test was used to determine differences in alpha diversity among different treatments. Significant differences between two treatments are marked with asterisks (*P< 0.05, **P< 0.01, ***P< 0.001). **(A, B)**: The Shannon index of bacterial and fungal communities in rhizosphere soils. **(C, D)**: The Shannon index of bacterial and fungal communities in roots of *S. miltiorrhiza*.

The rarefaction curves for bacteria and fungi exhibited a plateau, indicating that the sequencing depth was sufficient to capture most of the species present in both soil and root samples (**Figure S5**). For the rhizosphere soil microbial communities, the Shannon index of bacteria in the CF treatment significantly lower than that in the CK and TCMR treatments (**Figure 4A**, **Table S2**). All the TCMR treatments (excepted for MCRf) significantly reduced the Shannon index of the rhizosphere soil fungal communities compared to the CF treatments (**Figure 4B**, **Table S3**). For root microbial communities, the MCRf treatment significantly increased the Shannon index of bacteria (**Figure 4C**, **Table S4**); and all the TCMR treatments decreased the Shannon index of fungi to some extent compared to CK (**Figure 4D**, **Table S5**). Overall, MCRf exhibited the best effect on restoring the balance of the microbial community.

**Figure 5:**
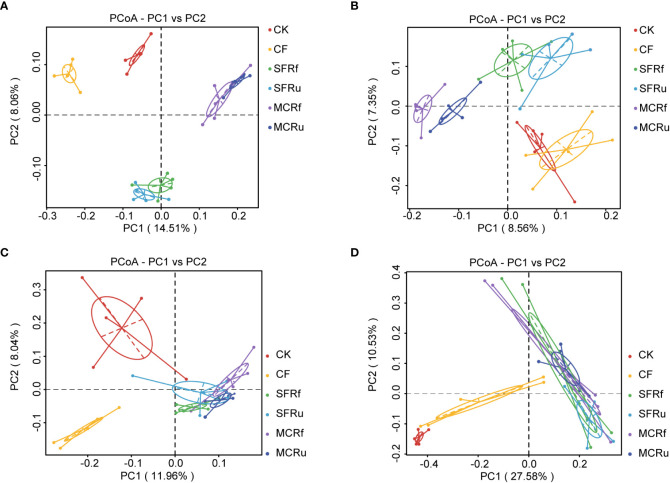
Effects of TCMRs on the beta diversity of the microbial communities in the rhizosphere soils and the roots of *S. miltiorrhiza* at harvest PCoA based on the unweighted UniFrac distance showing the differences in the compositions of the microbial communities among treatments. **(A, B)**: Bacterial and fungal communities in rhizosphere soils. **(C, D)**: Bacterial and fungal communities in roots of *S. miltiorrhiza*. Each point represents a sample, and samples from the same treatment are shown in the same color.

The PCoA and UPGMA results demonstrated that TCMRs altered the microbial community compositions of soils and roots. The bacterial and fungal compositions in the rhizosphere soils of the SFRf and SFRu treatments exhibited a high degree of similarity, as did those of the MCRf and MCRu treatments. However, they were obviously distinct from those observed in the CK and CF treatments (**Figures 5A, B**, **Figure S6A, B**). The bacterial and fungal community compositions in the roots among the four TCMR treatments did not exhibit significant differences, but they were all significantly different from those in the CK and CF treatments (**Figures 5C, D**, **S6C, D**).

#### 3.3.2 Changes in the composition of the bacterial and fungal communities of rhizosphere soils and roots of *Salvia miltiorrhiza*

At the phylum level, 61 bacterial phyla and 17 fungal phyla were identified in rhizosphere soils, and 54 bacterial phyla and 14 fungal phyla were identified in roots. The dominant bacterial phylum in the rhizosphere soil was Proteobacteria, with a significantly higher relative abundance in the four TCMR treatments compared to the CK and CF treatments (**Figures S7A, C**, **Table S6**). The dominant fungal phylum in the rhizosphere soil of all treatments was Ascomycota, with significantly higher relative abundance observed in the CF and SFRf treatments compared to the CK and SFRu treatments (**Figures S7B, D**, **Table S7**). Cyanobacteria was the dominant bacterial phylum in the root in CK, with significantly higher relative abundance than in the other treatments (**Figures S8A, C**, **Table S8**). Proteobacteria was the dominant bacterial phylum in the root in the CF and TCMR treatments, with significantly higher relative abundance in the TCMR treatments (except SFRu) compared to CK (**Figures S8A, C**, **Table S8**). Ascomycota was the dominant fungal phylum in the root in all treatments, with significantly higher relative abundance in the four TCMR treatments compared to the CK and CF treatments (**Figures S8B, D**, **Table S9**).

We focused on the changes in the composition of microbes at the genus level in rhizosphere soils and roots. The relative abundance of dominant genera differed among the various treatments. Compared to CK or CF, TCMRs resulted in an increase in the relative abundance of potential beneficial bacteria and a decrease in the relative abundance of pathogens.

**Figure 6:**
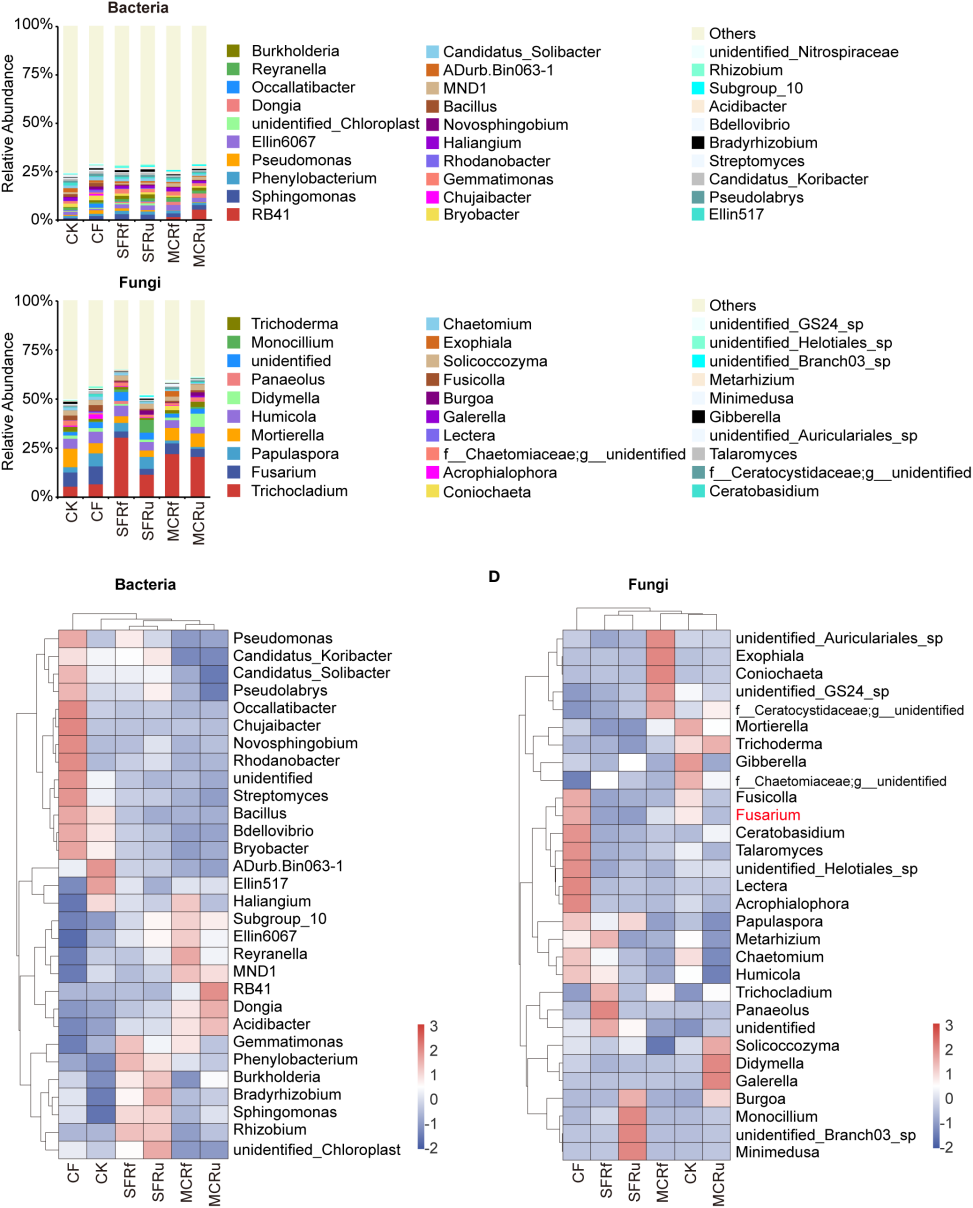
Effect of TCMRs on the microbial composition of the rhizosphere soils at the genus level **(A, B)**: Distribution and abundance of the top 30 genera of bacteria and fungi in rhizosphere soils. The category ‘others’ represents the sum of the relative abundances of all genera other than the 30 genera shown in the figure. **(C, D)**: Relative abundance clustering heatmap of the top 30 genera of bacteria and fungi in different treatments. The clustering tree on the left in the figure is the species clustering tree, and the clustering tree at the top of the figure is the sample clustering tree. The values corresponding to the heatmap are the Z scores obtained from the standardized relative abundances of species in each row. The legend shows the color intervals for the Z score. A higher relative abundance of a species in different treatments corresponds to a redder color, and a lower relative abundance corresponds to a bluer color.

For rhizosphere soils, each TCMR treatment significantly increased the relative abundance of some potential beneficial bacteria compared with CK, such as *Sphingomonas* and *Burkholderia-Caballeronia-Paraburkholderia* (abbreviated as *Burkholderia*) in the SFRf, SFRu, and MCRu treatments, *Bradyrhizobium* in the four TMCR treatments, and *Allorhizobium-Neorhizobium-Pararhizobium-Rhizobium* (abbreviated as *Rhizobium*) in the SFRf and SFRu treatments (**Figures 6A, C**, **Table S10**). Most of these genera were also significantly more abundant than in the CF treatment. In the rhizosphere soil fungal community, *Fusarium* was the most prevalent genus in the CF treatment, followed by the CK treatment (**Figures 6B, D**, **Table S11**), whose relative abundance in the CF treatment was 40.8-67.5% higher than that in the TCMR treatments (*P*<0.05). The relative abundance of potential beneficial fungi increased significantly in the TCMR treatments compared with CK, such as *Trichocladium* in all TCMR treatments and *Monocillium* in the SFRf, SFRu, and MCRu treatments (**Figures 6B, D**, **Table S11**). Most of these fungi were also significantly more abundant in these groups than in the CF treatment.

**Figure 7:**
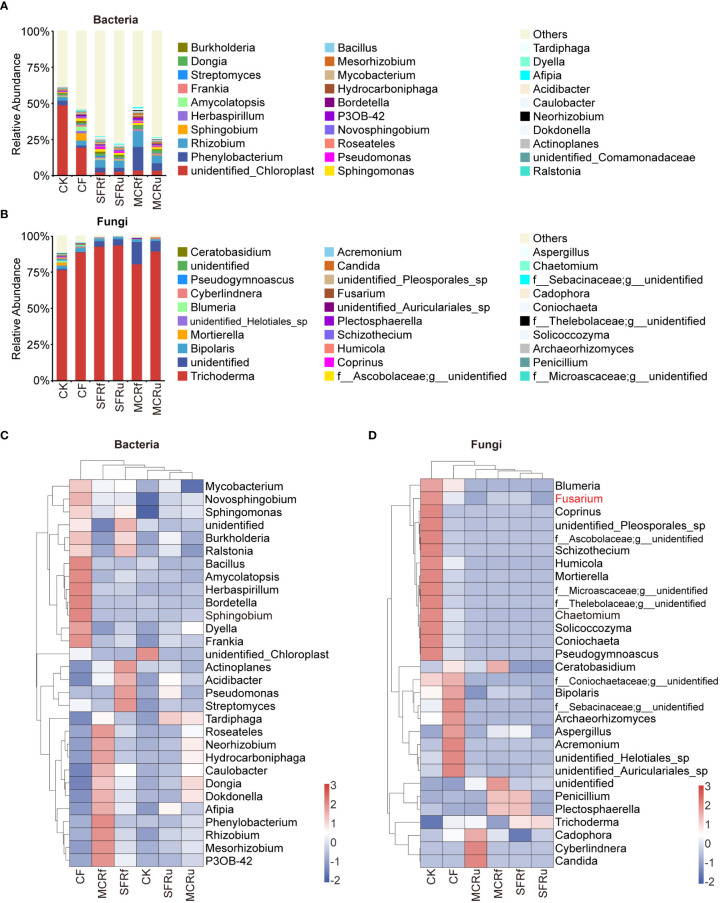
Effect of TCMRs on the microbial composition of the roots of *S. miltiorrhiza* at the genus level **(A, B)**: Distribution and abundance of the top 30 genera of bacteria and fungi in the roots of *S. miltiorrhiza*. The category ‘others’ represents the sum of the relative abundances of all genera other than the 30 genera shown in the figure. **(C, D)**: Relative abundance clustering heatmap of the top 30 genera of bacteria and fungi in different treatments. The clustering tree on the left in the figure is the species clustering tree, and the clustering tree at the top of the figure is the sample clustering tree. The values corresponding to the heatmap are the Z scores obtained from the standardized relative abundances of species in each row. The legend shows the color intervals for the Z score. A higher relative abundance of a species in different treatments corresponds to a redder color, and a lower relative abundance corresponds to a bluer color.

For root of *S. miltiorrhiza*, some potential beneficial bacteria also increased significantly in the TCMR treatments compared with CK or CF, such as *Sphingomonas* in all four TCMR treatments, *Pseudomonas* in the SFRf and SFRu treatments, *Rhizobium* in the MCRf treatment, and *Neorhizobium* in the MCRf and MCRu treatments (**Figures 7A, C**, **Table S12**). *Trichoderma* (67.43-98.57%) was the most abundant fungal genus in all roots (**Figures 7B, D**, **Table S13**), with significantly higher relative abundance in the SFRf, SFRu, and MCRu treatments compared to CK. In addition, three pathogens (*Fusarium*, *Bipolaris*, and *Blumeria*) were detected in roots, with significantly lower relative abundance in TCMR treatments compared to the CK or CF treatment (**Figures 7B, D**, **Table S13**).

#### 3.3.3 Analysis of significantly enriched species in rhizosphere soils and roots of *Salvia miltiorrhiza*

**Figure 8:**
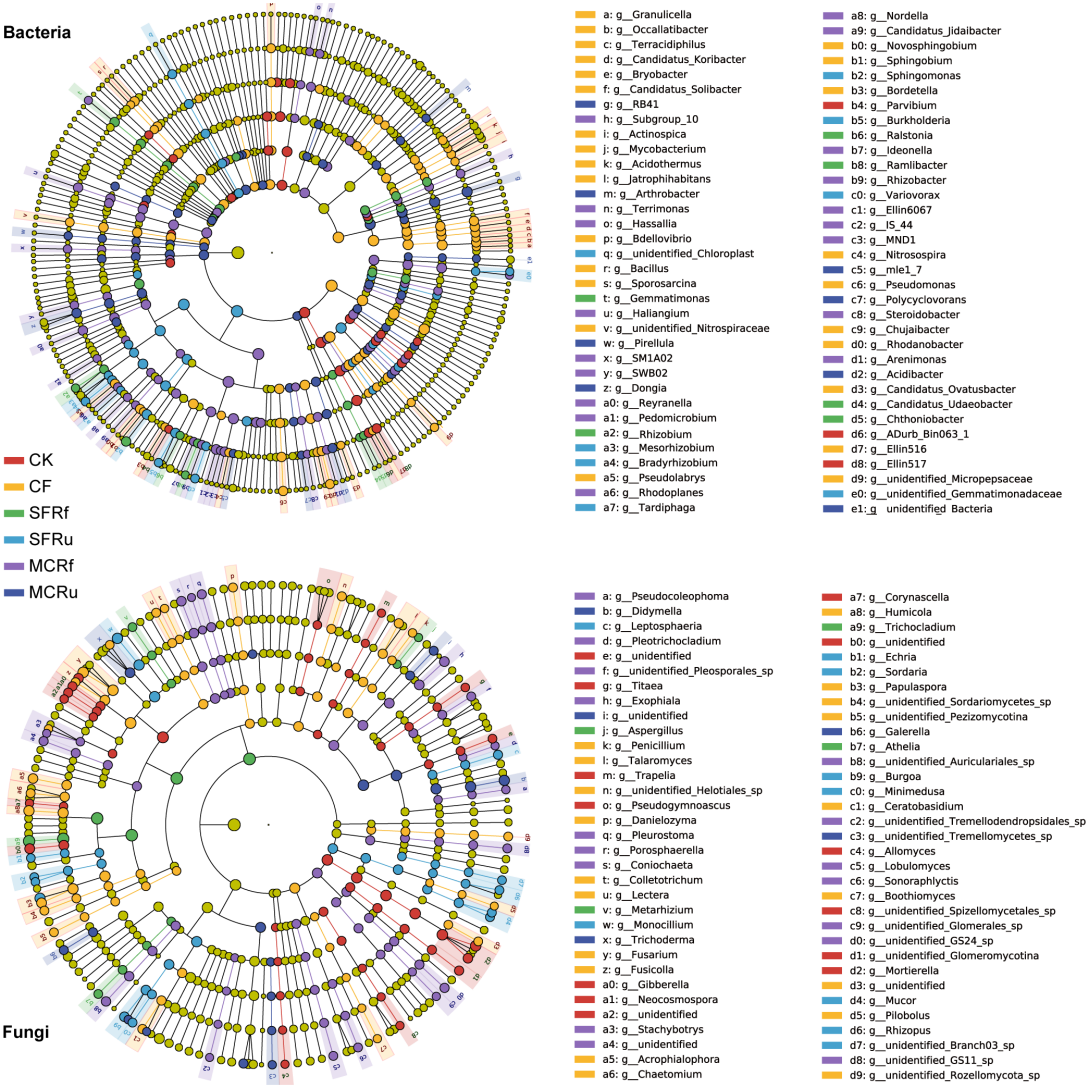
Effect of TCMRs on the enriched species in rhizosphere soils (LDA=3) In the LEfSe cladogram, the circles radiating from inside to outside represent the taxonomic level from phylum to species. Each small circle at different classification levels represents a classification at that level, and the diameter of each small circle is proportional to the relative abundance. Species with no significant differences are uniformly colored yellow, and the color of biomarkers is the same as that of the group. The legend shows the enriched species at the genus level.

LEfSe was employed to analyze the species that exhibited significant enrichment and played pivotal roles in different treatments. In the bacterial and fungal genera in the rhizosphere soils, 68 and 66 biomarkers were observed, respectively (**Figure 8**). In root bacterial and fungal genera, 36 and 23 biomarkers were observed, respectively (**Figure S9**).

We found that most potential beneficial bacteria in the top 30 genera in terms of relative abundance were enriched in the TCMR treatments (**Figure 8**, **S9**). The pathogen *Fusarium* was enriched in rhizosphere soils and roots in the CK and CF treatments (**Figure 8**, **S9**). In addition, some beneficial species with lower relative abundances (genera other than the top 30) were enriched in the TCMR treatments, such as *Ramlibacter* in rhizosphere soil in the SFRf treatment, *Rhizobacter* in rhizosphere soil in the MCRf treatment (**Figure 8**), and *Marinobacter* in roots in the MCRu treatment (**Figure S9**). These results indicated that beneficial bacteria with low abundance also facilitated the growth of plants in response to the TCMR treatments.

### 3.4 The relationship between *Fusarium* and the bacterial or fungal communities in rhizosphere soils

**Figure 9:**
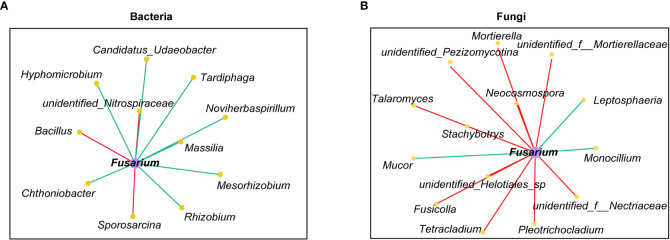
Correlation between *Fusarium* and bacterial and fungal communities **(A)** bacteria; **(B)** fungi, i.e. Relationships between bacterial and fungal communities (top 100 in terms of relative abundance) and *Fusarium* in soils were determined by calculating Spearman’s correlation coefficients. Bacteria and fungi with a correlation coefficient greater than 0.6 were used to construct the network (P< 0.05). Red and green lines indicate that the relative abundances of the bacteria and fungi were positively and negatively correlated, respectively, with the relative abundance of *Fusarium*.

A correlation analysis was performed to identify the bacterial and fungal genera (the top 100 in terms of relative abundance) closely associated with pathogenic *Fusarium* in rhizosphere soils. Most genera significantly negatively correlated with *Fusarium* were beneficial genera that increased or were enriched in the TCMR treatments, such as *Rhizobium*, *Mesorhizobium*, *Tardiphaga*, and *Monocillium* (**Figures 6**, **8**, **9**). In contrast, the genera significantly positively correlated with *Fusarium* were those increased or were enriched in the CK or CF treatments, such as *Bacillus, Talaromyces*, *Pezizomycotina*, and *Fusicolla* (**Figures 6**, **8**, **9**).

### 3.5 Comprehensive evaluation of the effect of TCMRs on promoting plant growth and improving soil health

The Z score was computed through standardization of diverse variables’ results, enabling comprehensive evaluation of the impact of various treatments on both *S. miltiorrhiza* yield and quality, as well as continuous monoculture soil health. The optimal treatment was determined by calculating the total score of different variables for each treatment. The ranking of the total score for yield and quality of *S. miltiorrhiza* from high to low is as follows: SFRf > MCRu > SFRu > MCRf > CK > CF, and that for soil health from high to low is as follows: SFRf > MCRf > MCRu > CK > SFRu > CF (**Tables 2**, **3**), indicating that SFRf have the significant impact on improving the growth and quality of *S. miltiorrhiza* as well as on soil health.

**Table 2:** Z scores of soil physicochemical properties and bacterial and fungal diversity in different treatments.

Treatment	Physicochemical properties				Bacterial diversity					Fungal diversity					*Fusarium* abundance	Total score
	pH	CEC	OM	C/N	Observed species	Shannon	Simpson	Chao1	ACE	Observed species	Shannon	Simpson	Chao1	ACE		
**CK**	0.923	-0.098	-0.149	-0.085	0.104	0.09	0.067	0.069	0.1	-0.152	-0.151	-0.094	-0.149	-0.139	-0.129	0.208
**CF**	-0.444	0.065	-0.159	-0.113	-0.253	-0.321	-0.237	-0.153	-0.168	-0.039	-0.198	-0.178	-0.006	-0.007	-0.258	-2.468
**SFRf**	-0.421	0.199	0.057	0.066	0.027	0.035	0.043	0.028	0.027	0.241	0.24	0.256	0.235	0.245	0.145	1.423
**SFRu**	-0.307	-0.19	-0.072	0.164	-0.129	-0.082	-0.061	-0.138	-0.142	0.148	0.053	-0.054	0.137	0.11	0.159	-0.403
**MCRf**	-0.148	0.075	0.216	-0.098	0.124	0.148	0.158	0.143	0.145	-0.123	-0.012	0.023	-0.129	-0.119	-0.006	0.398
**MCRu**	0.399	-0.052	0.106	0.067	0.126	0.129	0.103	0.05	0.038	-0.075	0.067	0.037	-0.088	-0.091	0.08	0.896

CEC, cation exchange capacity; OM, organic matter; C/N, ratio of C to N. The lower the fungal diversity and the abundance of pathogens are; the healthier the soil. Therefore; the opposite numbers of the Z scores of fungal diversity and the abundance of pathogens are displayed in the table.

## 4 Discussion

### 4.1 Application of TCMRs improves the physicochemical properties of continuous monoculture soils

Healthy soil is rich in organic matter which allows a high diversity of soil organisms to flourish and acts as a reservoir of soil nutrients and moisture (Turmel et al., 2015). Continuous monoculture leads to soil degradation, including a decrease in OM content. TCMRs are rich in nutrients and beneficial for soil improvement (Ma et al., 2019a; Lu and Li, 2021). The application of TCMRs has shown a significant increase in the OM content and CEC of soils (Ma et al., 2019a; Ma et al., 2019b). In this study, the SFRf, MCRf, and MCRu treatments significantly increased the soil OM content (**Figure 3**). This observed increase can be attributed to the substantial amount of carbon (C) present in TCMRs (**Table S1**), which serves as a crucial source of soil C and subsequently increases the amount of soil OM. Soil OM is the most significant indicator of soil health (Nunes et al., 2020). It improves soil fertility by providing cation exchange sites and serves as a reserve store of plant nutrients (Karlen et al., 1997; Wander et al., 2019). Adding OM can also increase soil aggregation. Soil aggregates and their stability influence soil porosity, movement of water, gas, and nutrients through the soil system, and root development (Verhulst et al., 2010). The SFRf treatment significantly increased the soil CEC (**Figure 3A**) which is the capacity of the soil particles to hold cations for exchange with the soil solution and is an indicator of soil fertility (Moebius-Clune et al., 2016). An increase in CEC is caused by an increase in OM content (Soares and Alleoni, 2008) because OM contains many acidic functional groups, the dissociation of which may increase the soil negative charge and subsequently increase soil CEC (Shi et al., 2019). A soil C/N ratio of less than ten is detrimental to soil quality and good crop growth (Magdoff et al., 1997). Our results showed that the C/N ratio of soil in the CK and CF treatments was less than ten and lower than that in all TCMR treatments at harvest (**Figure 3**). Additionally, despite having the highest level of available N, P, and K content at harvest in soils (**Figure 3**), the CF treatment did not exhibit higher nutrient accumulation in plants compared to the TCMR treatments, except for the N accumulation in roots (**Figure 1**). This means that the nutrients provided by TCMRs are capable of satisfying the requirement of the plant. This result is associated with the release rates of nutrients from diverse fertilizers. The nutrients in CF are rapidly released in soils, resulting in a high level of available nutrients. While OM must be converted into available nutrients by microbial degradation (Doan et al., 2015; Xin et al., 2017), which is a slow process, and the released available nutrients are constantly consumed by the plants, resulting in lower levels of available nutrients in TCMR treatments than that in the CF treatment. In general, the SFRf treatment exhibited the most significant impact on enhancing the physicochemical properties of the continuous monoculture soil by increasing the OM content, CEC, and C/N.

### 4.2 Application of TCMRs improves the microbial community structure of continuous monoculture soils and roots

#### 4.2.1 TCMRs increase bacterial diversity

The soil bacterial community plays an important role in soil nutrient cycling, and its diversity has become the key indicator for evaluating soil health (Hermans et al., 2017). Applying CF in continuous cropping soil decreased the soil bacterial diversity (Shen et al., 2010). Our results showed that the application of CF significantly reduced the bacterial diversity of rhizosphere soil at harvest compared to CK (**Figure 4**). In contrast, although all TCMR treatments significantly reduced the bacterial diversity in the soil before planting (**Figure S10**) due to the residual active ingredients, the TCMR treatments restored the bacterial diversity in rhizosphere soil at harvest to the same level of CK and significantly higher than that in the CF treatment; even the MCRf treatment significantly increased the bacterial diversity in roots (**Figure 4**). Resource partitioning is a crucial mechanism for preserving the microbial diversity of soil ecosystems (Hanson et al., 2008). The input of external OM provides more nutrients, enabling the recruitment of additional functional complementary microbial species to maintain the microbial diversity of continuous monoculture soil (Sun et al., 2016; Shu et al., 2022). Other studies have demonstrated that incorporating plant residues into the soil increases soil temperature and aeration, thereby creating favorable conditions for microbial growth and activity (Turmel et al., 2015). Furthermore, Zhang et al. (2023) reported that the OM content had a strong effect on the network complexity of sensitive bacterial taxa. In this study, co-occurrence network analysis demonstrated that the bacterial networks of the TCMR treatments had higher node numbers, edge numbers, and average degrees than those of the CK and CF treatments, which showed that the TCMR treatments produced a more complex and stable bacterial community (**Figure S11**). These findings suggested that the application of TCMR increased bacterial diversity and activity and formed a more complex and stable bacterial community in continuous cropping soil and roots compared with the effect of CF.

#### 4.2.2 TCMRs recruit beneficial microbes

The application of plant residues markedly influences the soil microbial community, forming a significant group compared to unamended soils (Lian et al., 2019), promoting the enrichment of beneficial microbes and the mortality of pathogenic microbes (Cobo-Díaz et al., 2022). In the present study, the relative abundances of some beneficial bacteria increased in rhizosphere soils and roots in the TCMR treatments, such as *Sphingomonas*, *Burkholderia*, *Rhizobium*, *Bradyrhizobium*, *Trichoderma*, and *Monocillium* (**Figures 6**, **7**, **8** and **S9**). Some species from these genera have capacities to produce auxin, carry iron and dissolve phosphorus, thereby playing a pivotal role in promoting plant growth, abiotic stress resistance, bioremediation, and biodegradation (Rhijn and Vanderleyden, 1995; Halo et al., 2015; Sallam et al., 2019; Hwang et al., 2021). We observed that beneficial microbial genera, whose relative abundance increased in the TCMR treatments, were positively correlated with the growth of *S. miltiorrhiza* (**Figure S12**). Conversely, the genera that showed an increase in relative abundance in the CF treatment were negatively correlated with the quality of *S. miltiorrhiza* (**Figure S12**). We subsequently isolated strains of *Burkholderia*, *Rhizobium*, and *Sphingomonas* from *S. miltiorrhiza* roots and rhizosphere soils. Our unpublished data confirmed that some of these strains promote plant growth by producing indole acetic acid (IAA) and dissolving phosphorus. It will be worthwhile to reintroduce these beneficial bacteria into the soil to investigate how they affect the growth and disease control of *S. miltiorrhiza* in continuous cropping in future studies. Additionally, rhizosphere microbiota is influenced by root exudates (Bais et al., 2006). Bao et al. (2021) found that tanshinones have a repelling effect on Bacilli while specifically attracting Betaproteobacteria and Alphaproteobacteria in soils. In this study, the TCMR treatments resulted in significantly higher tanshinones accumulation in roots compared to CF. This compound may be secreted into the rhizosphere soils, leading to a significant increase of Proteobacteria in TCMR treatments. Further research is necessary to elucidate the deeper mechanism of TCMRs recruiting beneficial bacteria.

#### 4.2.3 TCMRs inhibit pathogens

The pathogens for root rot in *S. miltiorrhiza* belong to *Fusarium*, such as *F. oxysporum*, *F. equiseta*, and *F. solani* (Wang et al., 2018b; Li et al., 2022; Pu et al., 2022). In the present study, all the TCMR treatments significantly reduced the relative abundance of *Fusarium* in both rhizosphere soils and roots, as well as the incidence and disease index of root rot in *S. miltiorrhiza*. The CF treatment did not significantly affect these parameters (**Figures 6**, **7**, **Figure S1**). A recent study also found a 54% reduction in *F. oxysporum* in the TCMR treatment versus the control (Zhou et al., 2023). Two potential explanations are proposed for the decrease in the relative abundance of *Fusarium* after the application of TCMRs. One is that multiple active substances remain in SFR and MCR (**Figure S13**), which may exert an inhibitory effect on pathogenic *Fusarium*. The other is that the relative abundance of some beneficial bacteria significantly increased in the soils of the TCMR treatments, including *Burkholderia*, *Sphingobium*, and *Rhizobium*, which were significantly negatively correlated with the abundance of *Fusarium* (**Figure 9**), suggesting that they may inhibit the growth of *Fusarium*. *Burkholderia* is the most commonly reported genus of bacteria with antagonistic activity against *Fusarium* and inhibits *Fusarium* diseases in many plants, including maize, tomatoes, and bananas (Abdel-Aziz et al., 2020; Barrera-Galicia et al., 2021). Beneficial microorganisms may shield plants from diseases by altering the plant immune system (Liu et al., 2021). In this way, TCMRs suppressed pathogens through residual active ingredients and the recruitment of beneficial microbes.

### 4.3 TCMRs are better than CF in increasing the yield and active ingredient accumulation in *Salvia miltiorrhiza*

Soil health is a crucial factor that impacts plant growth. Our findings indicated that applying CF and TCMRs promoted the growth of *S. miltiorrhiza*, with the yield improvement effect of the SFRf treatment surpassing that of the CF treatment by a considerable margin (**Table 3**). The shoot and root biomass of the SFRf treatment increased significantly by 41.05% and 31.82%, respectively, compared with the CF treatment (**Figure 1**). This result does not appear to be related to the available inorganic nutrient contents at harvest in the soil, as the CF treatment soil had significantly higher available N, P, and K contents than the SFRf treatment soil (**Figure 3**). Alyokhin et al. (2005) found that the OM and microbial activity in soils provide a buffering capability, which helps to maintain nutrient balance in plants and promotes optimal plant growth. In this study, compared to the CF treatment, the SFRf treatment increased the OM content and CEC of the soil (**Figure 3**), as well as the microbial activity of the roots (**Figure 4C**). The SFRf treatment also recruited beneficial microbes in soils and roots which promoted plant growth and decreased the relative abundance of pathogenic *Fusarium*. Thus, SFRf is better than CF at increasing the yield of *S. miltiorrhiza* by improving the health of continuous monoculture soils.

**Table 3:** Z scores of biomasses and active ingredients of *S. miltiorrhiza* in different treatments.

Treatment	Biomass		Active ingredients										Total score
	DW_s	DW_r	SvaB_s	Rsa_s	Cfa_s	SvaB_r	Rsa_r	Cfa_r	DTsn_r	CTsn_r	TsnI_r	TsnIIA_r	
**CK**	-0.104	-0.049	-0.047	-0.034	-0.067	-0.028	0.020	-0.028	-0.009	0.016	0.005	0.018	-0.307
**CF**	-0.023	-0.039	0.004	-0.032	0.014	-0.088	-0.094	-0.116	-0.157	-0.159	-0.176	-0.167	-1.033
**SFRf**	0.107	0.084	0.108	0.078	0.027	0.105	0.044	0.026	0.037	0.033	0.044	0.040	0.733
**SFRu**	0.075	-0.007	0.033	0.055	0.063	-0.018	-0.055	-0.020	0.017	0.024	0.045	0.041	0.254
**MCRf**	-0.010	0.002	-0.050	-0.035	-0.007	-0.006	0.013	0.051	0.026	0.030	0.027	0.034	0.076
**MCRu**	-0.044	0.010	-0.051	-0.032	-0.031	0.034	0.071	0.087	0.086	0.056	0.055	0.035	0.275

DW, dry weight; Cfa, caffeic acid; Rsa, rosmarinic acid; SvaB, salvianolic acid B; DTsn I, dihydrotanshinone I; CTsn, cryptotanshinone; Tsn I, tanshinone I and Tsn IIA, tanshinone IIA. The postfix ‘s’ represents ‘shoot’; and the postfix ‘r’ represents ‘root’.

We observed that CF significantly decreased the accumulation of seven active ingredients in roots compared with CK (**Figure 2**), implying that fertilization practices used in actual production were not conducive to improving *S. miltiorrhiza* quality. The accumulation of seven active ingredients in the roots in the four TCMR treatments was significantly greater in the CF treatment (**Figure 2**). Previous studies have shown that excessive nitrogen application reduced tanshinone accumulation in the roots of *S. miltiorrhiza* (Wang et al., 2011; Shi et al., 2021), which is due to the imbalance of mineral nutrients in plants. The active ingredients are the final products synthesized from chemical precursors, such as simple sugars and free amino acids in plants. Nutrient imbalances in plants can lead to reduced effectiveness of biochemical pathways, resulting in decreased accumulation of precursors and active ingredients (Phelan et al., 1996). In this study, the increase in the N accumulation in the roots in the CF treatment (101.7%) was significantly higher than that in the TCMR treatments (17.0-29.1%), which might lead to excessive nitrogen in plants (**Figure 1B**), resulting in imbalanced mineral nutrients and decreased accumulation of active ingredients.

## 5 Conclusions

Continuous monoculture of crops leads to reduced yield and quality and deteriorated soil quality. Our study demonstrated that TCMRs increased the yield and quality of *S. miltiorrhiza* by improving the health of continuous monoculture soil, including improving soil physicochemical properties and restoring the balance of the microbial community structure. The application of SFRf exerted the best effect, indicating that it has the most potential to entirely or partially replace CF in *S. miltiorrhiza* production. More research is required to investigate the composting process of TCMRs, the effects of beneficial strains enriched by TCMRs on *S. miltiorrhiza*, and the construction of the synthetic functional microbial community.

## Data availability statement

The original contributions presented in the study are publicly available. This data can be found here: NCBI-SRA repository, accession number PRJNA913695.

## Author contributions

SL: Data curation, formal analysis, investigation, software, validation, visualization, and writing - original draft. GY: Writing - review & editing. FW: Writing - review & editing and formal analysis. YG: Writing - review & editing. FLiu: Investigation and data curation. CP: Investigation and writing - review & editing. ZW: Investigation. YS: Writing - review & editing. XZ: Writing - review & editing. YL: Investigation. FLi: Investigation. YZ: Investigation. MC and LH: Conceptualization, funding acquisition, methodology, supervision, project administration, resources and writing - review & editing. All authors contributed to the article and approved the submitted version.
