# Supplementary material for: Traditional Chinese medicine residues promote the growth and quality of Salvia miltiorrhiza Bunge by improving soil health under continuous monoculture
Source: Front Plant Sci. 2023 Jun 7;14:1112382. doi: 10.3389/fpls.2023.1112382 (PMC10284172; doi:10.3389/fpls.2023.1112382)
Supplement: Supplementary file 1 [file DataSheet_1.docx]

Supplementary Material

Traditional Chinese medicine residues promote the growth and quality of *Salvia miltiorrhiza* Bunge by improving soil health under continuous monoculture

Sha Liu^1,2,3^, Guang Yang^2^, Faming Wu^3^, Yang Ge^2^, Fusong Liu^3^, Chunjuan Pu^2^, Zihan Wang^2^, Ye Shen^2^, Xiuteng Zhou^2^, Yuzhi Luo^2^, Fengsheng Li^4^, You Zhang^4^, Meilan Chen^2^*, Luqi Huang^2^*

^1^School of Pharmacy, Chengdu University of Traditional Chinese Medicine, Chengdu 611137, Sichuan, China

^2^National Resource Center for Chinese Materia Medica, China Academy of Chinese Medical Sciences, Beijing 100700, China

^3^School of Pharmacy, Zunyi Medical University, Zunyi 563000, Guizhou, China

^4^ Cultivation Base Department, Laiwu Purple Light Ecological Park Co., Ltd., Jinan 250022 Shandong, China

*** Correspondence:**Meilan Chen; Luqi Huang
chenmeilan@nrc.ac.cn; huangluqi01@126.com


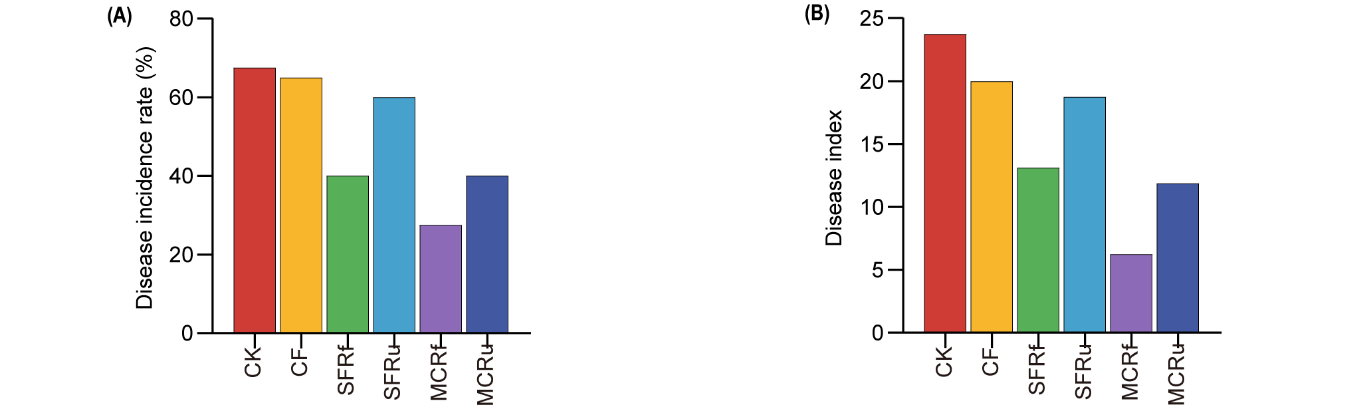


**Figure S1.** **Effect of TCMRs on the incidence of root rot of *S. miltiorrhiza*.** (A): Disease incidence rate of root rot in different treatments. (B): The disease index of root rot in different treatments. CK: no fertilizer; CF: chemical fertilizer; SFRf: fermented *Sophora flavescens* radix residue; SFRu: unfermented *Sophora flavescens* radix residue, MCRf: fermented *Moutan cortex* residue; MCRu: unfermented *Moutan cortex* residue.


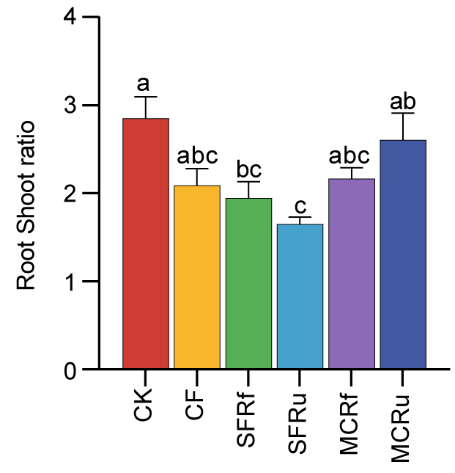


**Figure S2. Effect of TCMRs on the root shoot ratio of *S. miltiorrhiza*.** The error bar above each column represents the SEM value. Different letters indicate significant differences between treatments at *P* < 0.05. (n=13)


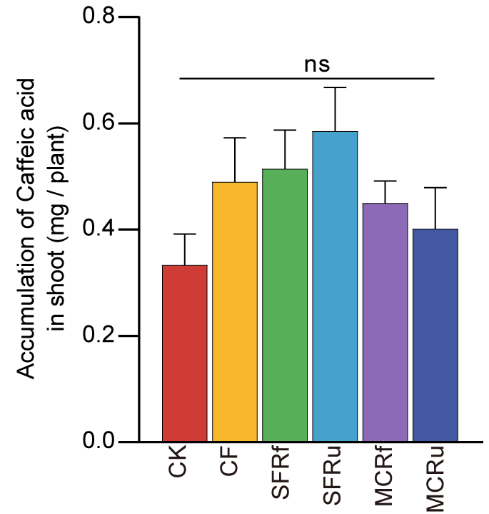


**Figure S3. Effect of TCMRs on the accumulation of caffeic acid in shoots of *S. miltiorrhiza*.** The error bar above each column represents the SEM value. ‘ns’ indicates that the difference among different treatments is not significant. (n=13)


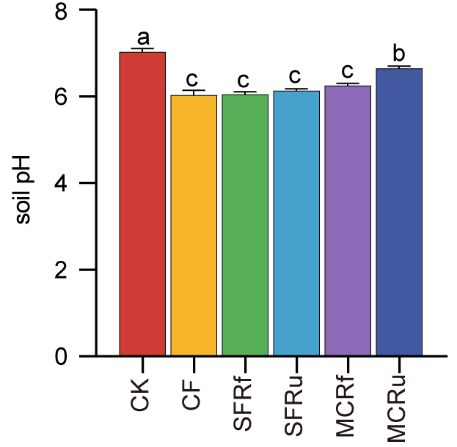


**Figure S4.** **Effect of TCMRs on the soil pH at harvest.** The error bar above each column represents the SEM value. Different letters indicate significant differences between treatments at *P* < 0.05. (n=6).
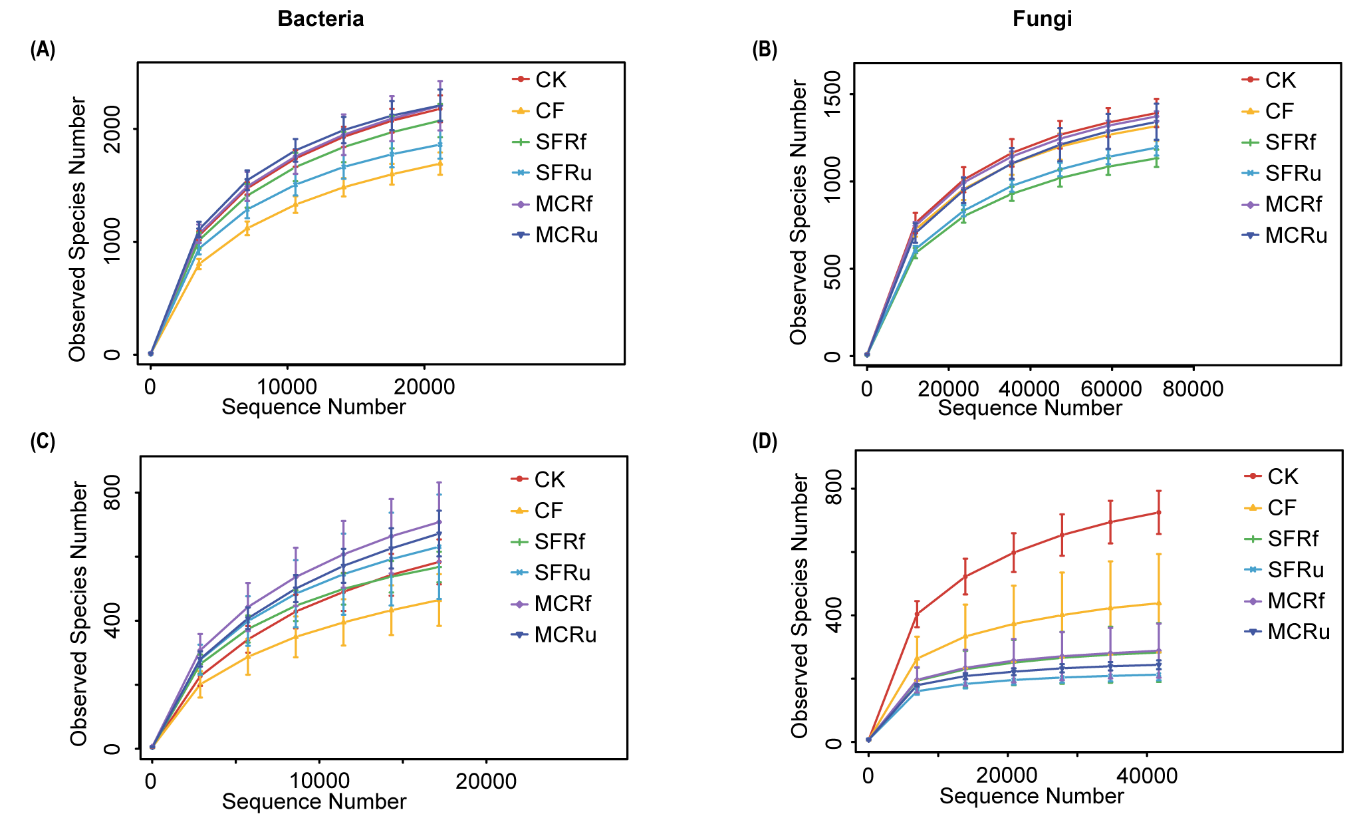


**Figure S5. The rarefaction curve of bacteria and fungi in different treatments.** (A), (B): The rarefaction curve of the bacteria and fungi in rhizosphere soils. (C), (D): The rarefaction curve of the bacteria and fungi in roots of *S. miltiorrhiza*. The abscissa shows the number of sequences randomly selected from a sample, and the ordinate shows the number of species that can be observed in those sequences, which is used to show the depth of sequencing.


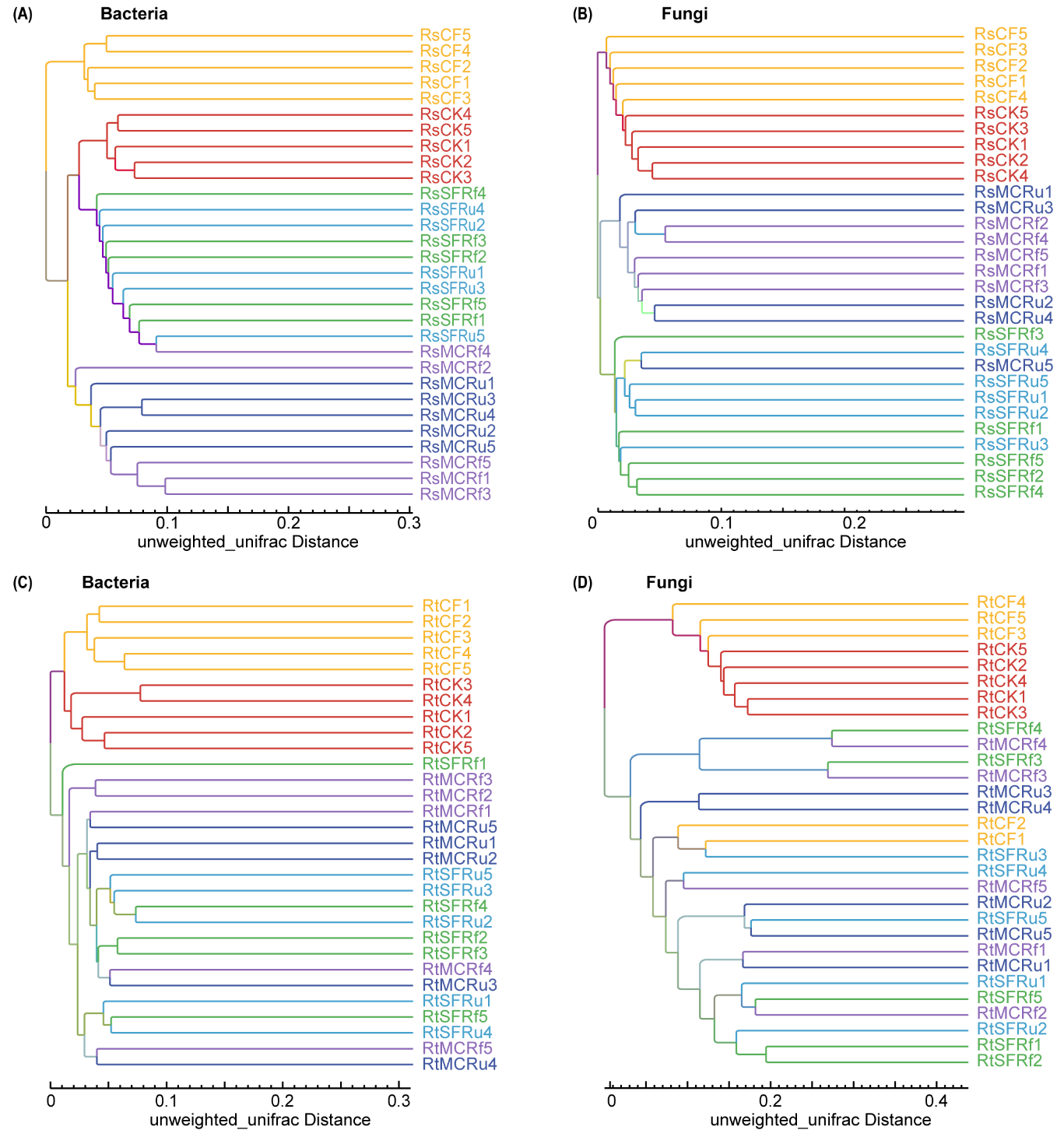


**Figure S6. The UPGMA cluster trees of the microbial communities.** The cluster tree reflects the similarity in different samples in the same treatment. (A), (B): The cluster tree of bacteria and fungi in rhizosphere soils. (C), (D): The cluster tree of bacteria and fungi in the roots of *S. miltiorrhiza*.

**
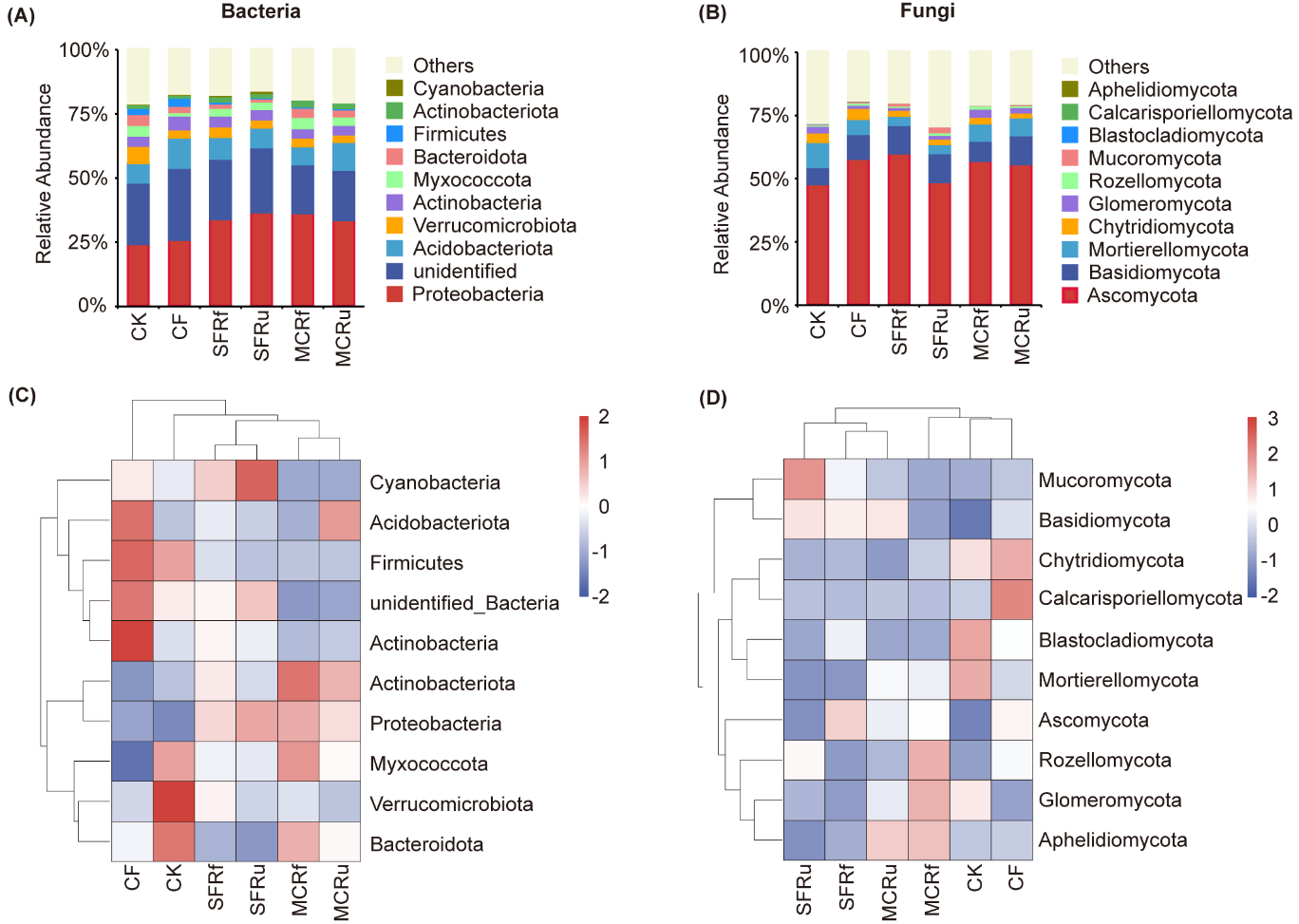
**

**Figure S7.** **Effect of TCMRs on the microbial composition of rhizosphere soils at the phylum level.** (A), (B): Distribution and abundance of the top 10 phyla of bacteria and fungi in different treatments. The category ‘others’ represents the sum of the relative abundances of all phyla other than the 10 phyla shown in the figure. (C), (D): Relative abundance clustering heatmap of the top 10 phyla of bacteria and fungi in different treatments. The clustering tree on the left in the figure is the species clustering tree, and the clustering tree on the top of the figure is the sample clustering tree. The values corresponding to the heatmap are the Z scores obtained from the standardized relative abundances of species in each row. The legend shows the color intervals for the Z score.


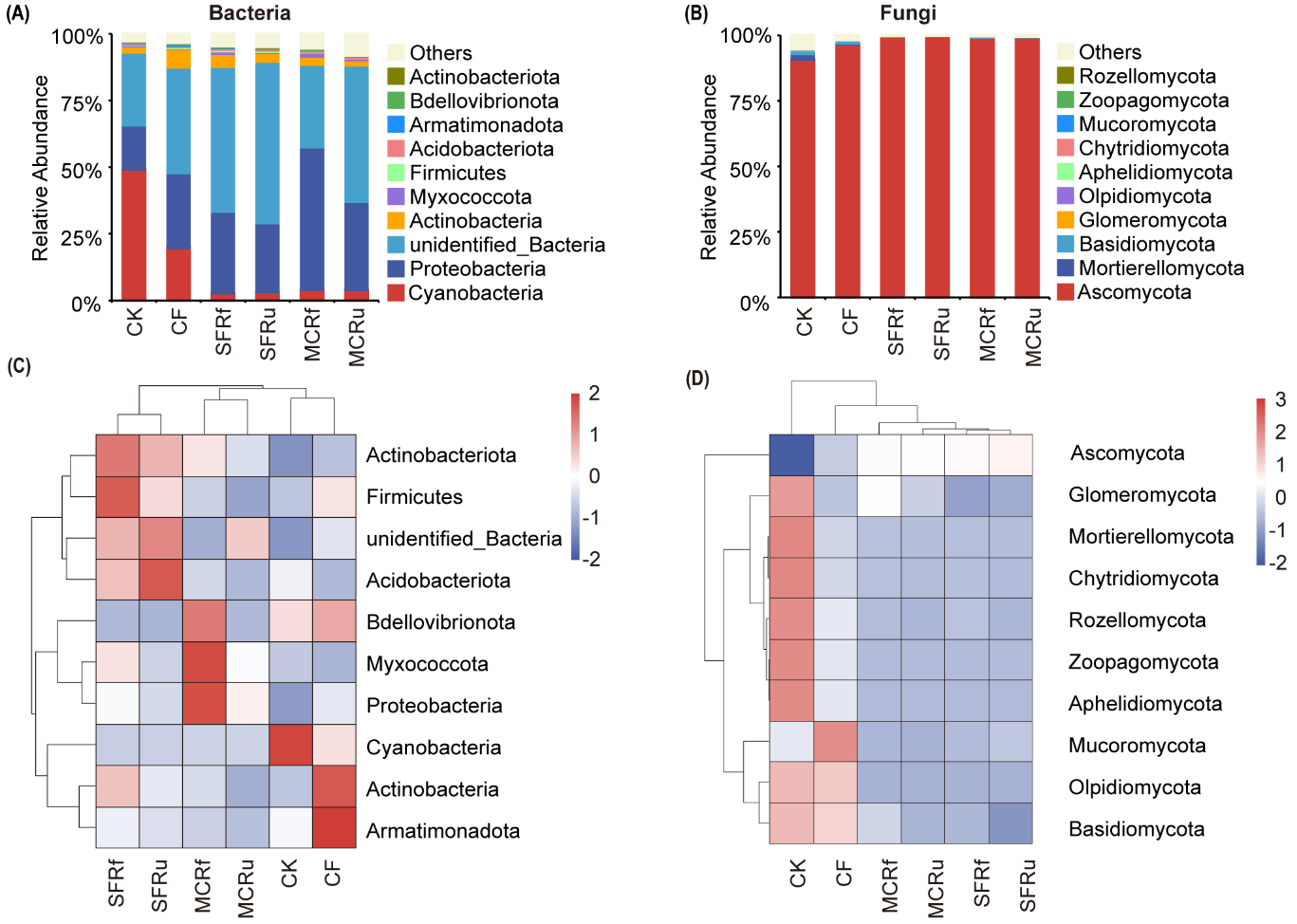


**Figure S8. Effect of TCMRs on the microbial composition in roots of *S. miltiorrhiza* at the phylum level.** (A), (B): Distribution and abundance of the top 10 phyla of bacteria and fungi in different treatments. The category ‘others’ represents the sum of the relative abundances of all phyla other than the 10 phyla shown in the figure. (C), (D): Relative abundance clustering heatmap of top 10 phyla of bacteria and fungi in different treatments. The clustering tree on the left in the figure is the species clustering tree, and the clustering tree on the top of the figure is the sample clustering tree. The values corresponding to the heatmap are the Z score obtained from the standardized relative abundances of species in each row. The legend shows the color intervals for the Z score.


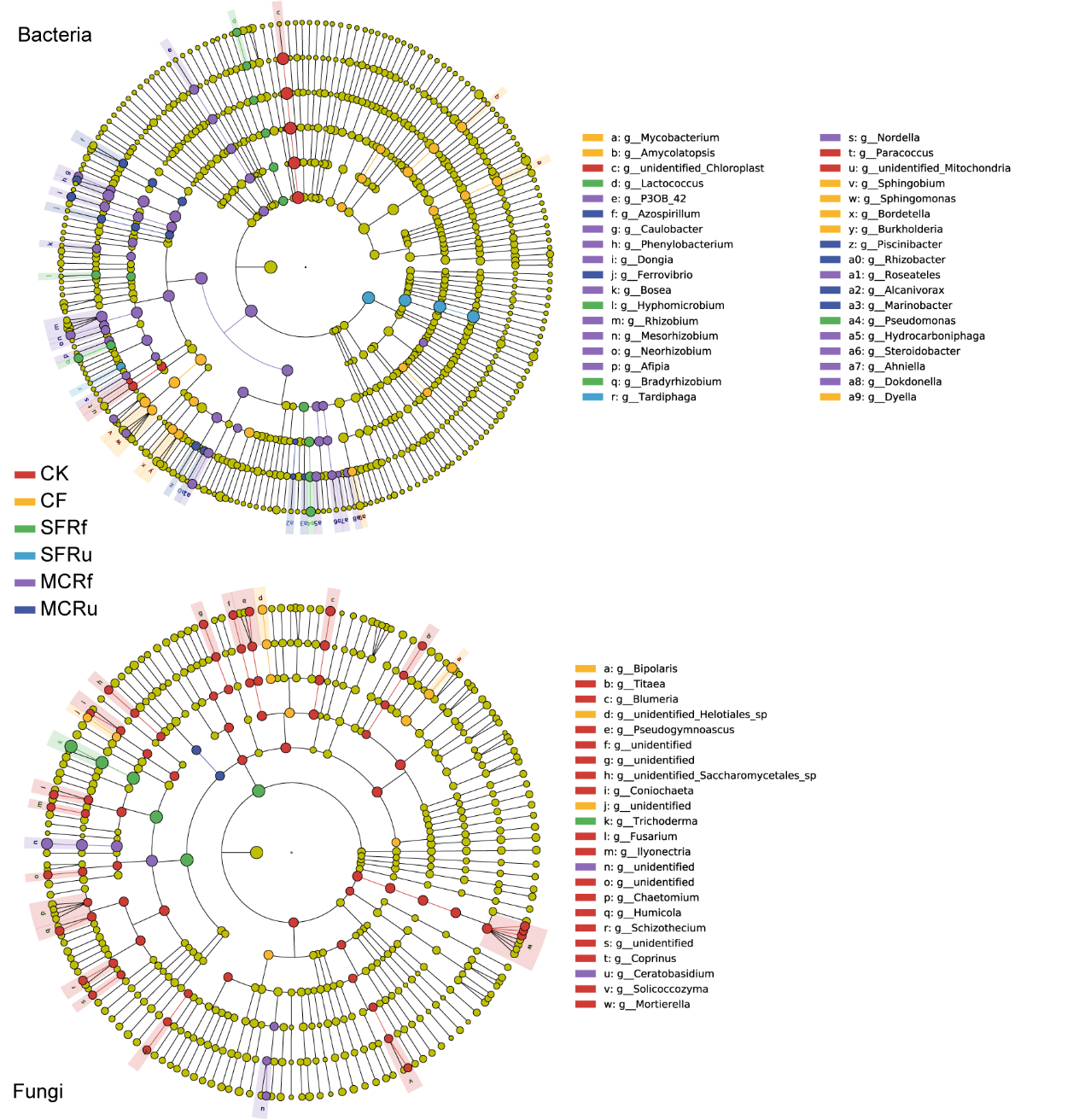


**Figure S9. The enriched species of *S. miltiorrhiza* roots in different treatments.** (LDA=3). In the LEfSe cladogram, the circles radiating from inside to outside represent the taxonomic level from phylum to species. Each small circle at different classification levels represents a classification at that level, and the diameter of each small circle is proportional to the relative abundance. Species with no significant differences are uniformly colored yellow, and the color of biomarkers is the same as that of the group. The legend shows the enriched species at the genera level.


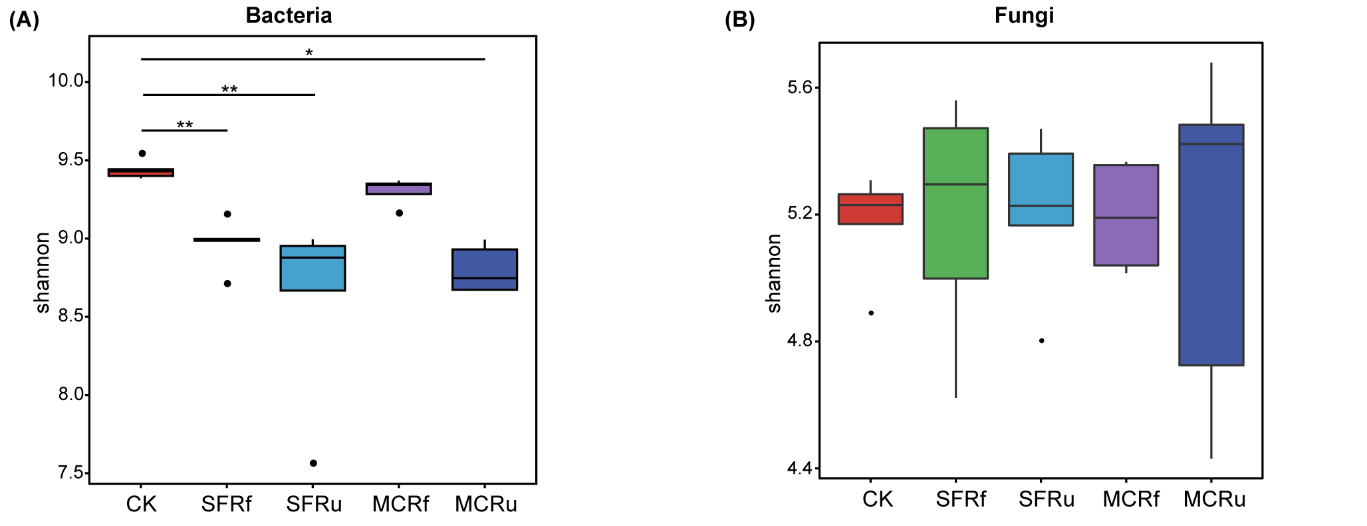


**Figure S10. The microbial alfa diversity of soils before planting.** The Shannon index indicates the microbial diversity in each treatment. The box chart shows the median, maximum, minimum, and abnormal values within the group. The higher the Shannon index is, the higher the diversity of the community. The Tukey’s test was used to determine differences in alpha diversity among different treatments. Significant differences between two treatments are marked with asterisks (* *P* < 0.05, ** *P* < 0.01).


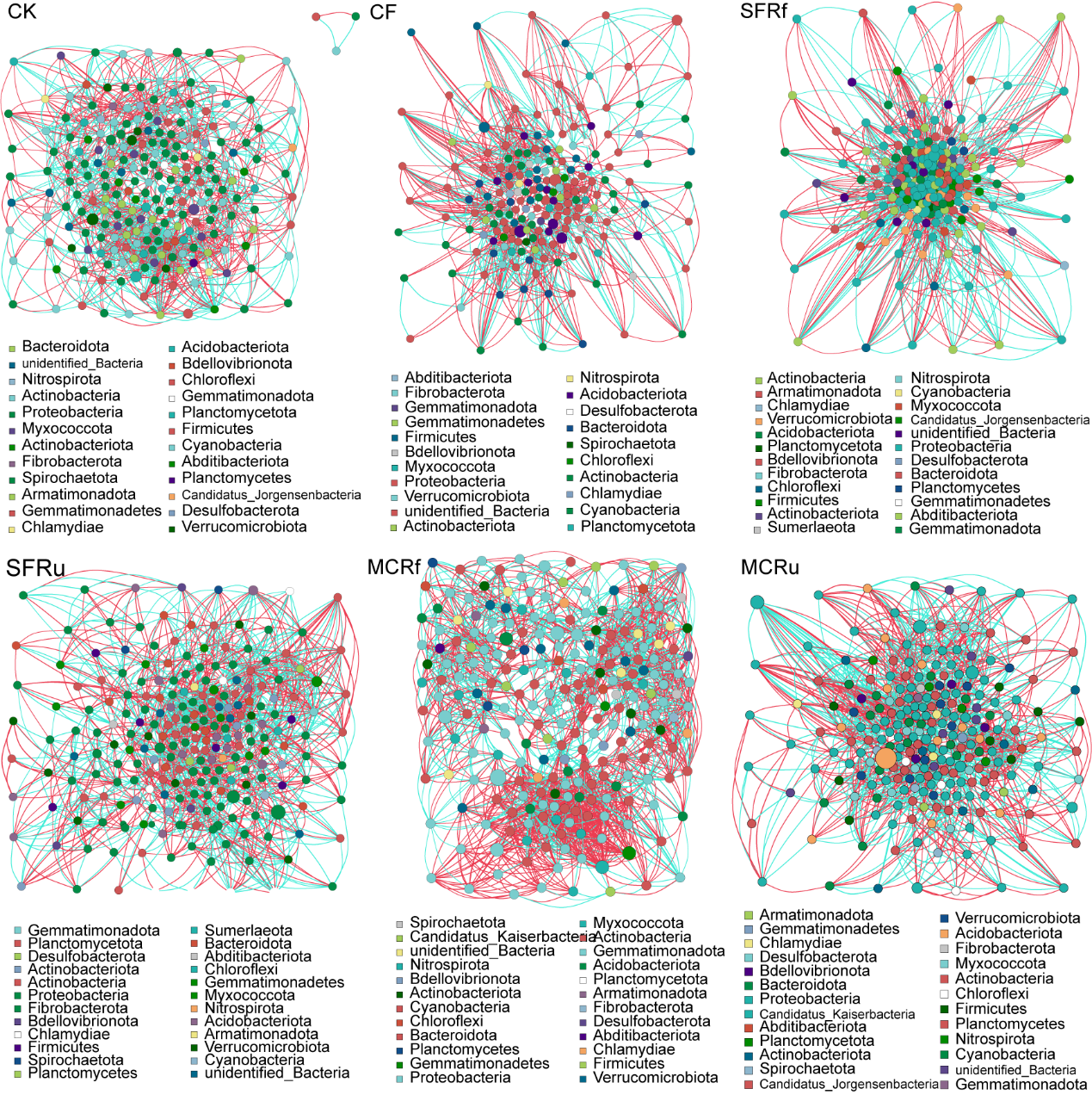


**Figure S11. The Co-occurrence networks analysis of the bacterial community in rhizosphere soils at harvest.** Network diagrams are constructed using bacteria genera as nodes and Spearman’s correlation coefficients values as edges. The bacterial genera are filtered according to the following rules: removing the connections with correlation coefficients lower than 0.6, eliminating self-connections of nodes, and excluding connections with node abundance less than 0.005%. Different nodes represent different genera, node size represents the average relative abundance of each genus. Nodes belonging to the same phylum are color-coded identically (as shown in the legend), and the thickness of each line is proportionate to Spearman's correlation coefficient. The red and blue lines denote positive and negative correlation, respectively.


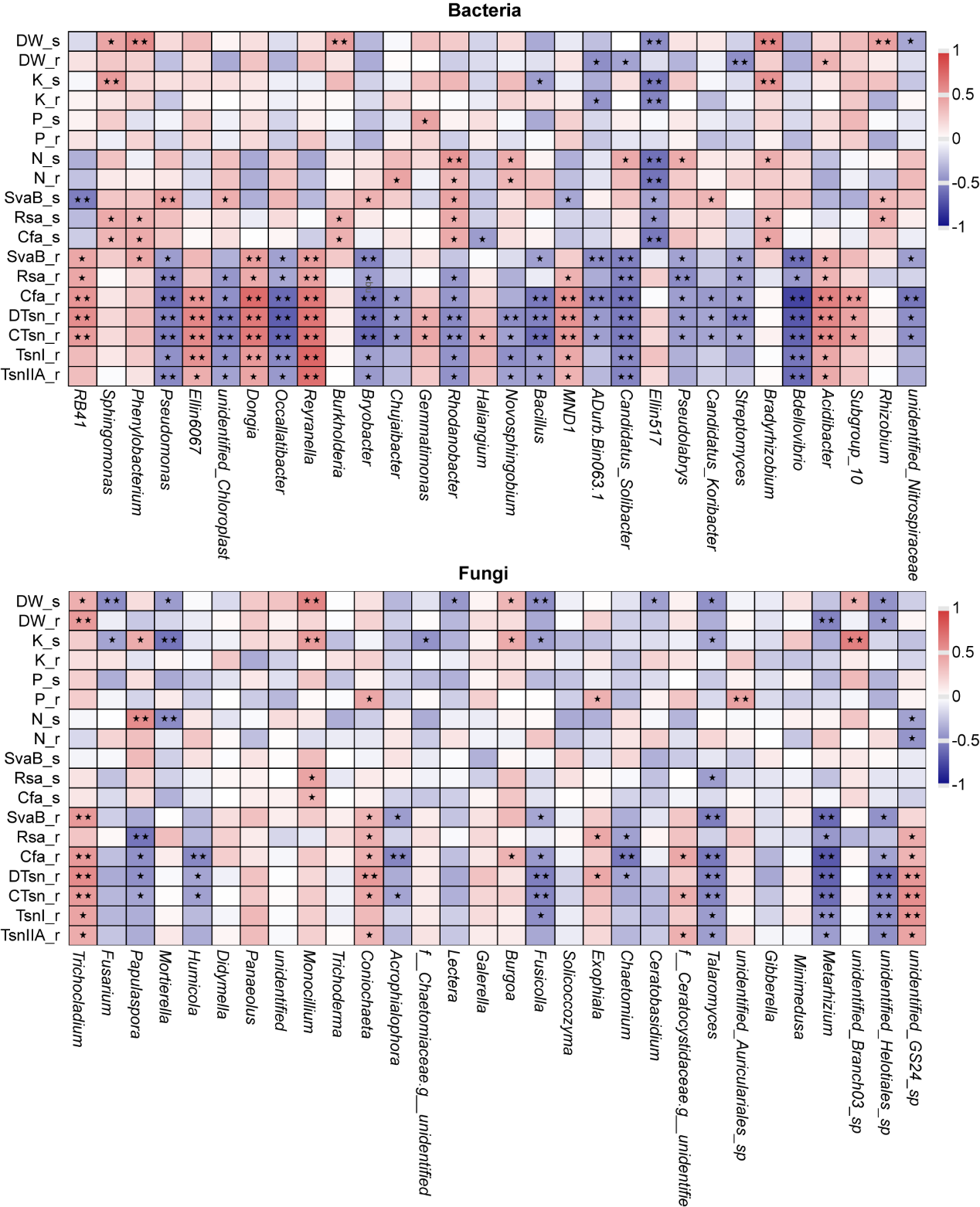


**Figure S12. The heatmap of the correlation between the top 30 bacterial and fungal genera in rhizosphere soils and the *S. miltiorrhiza* growth and quality.** The bacterial and fungal genera are shown at the bottom of the picture, the indicators of growth and quality of *S. miltiorrhiza*. are shown on the left of the picture. Dw_s and Dw_r: dry weight of plant tissues (postfix ‘s’ represents ‘shoot’, postfix ‘r’ represents ‘root’, the same later). N, P, K: accumulation of N, P, K per plant. Cfa: caffeic acid, Rsa: rosmarinic acid, SvaB: salvianolic acid B, DTsn: dihydrotanshinone I, CTsn: cryptotanshinone, Tsn I: tanshinone I, Tsn IIA: tanshinone IIA. Spearman method was used for correlation analysis. The legend represents the color intervals for different R values. The number of asterisks indicates the degree of correlation, *P* < 0.05: *, *P* < 0.01: **.


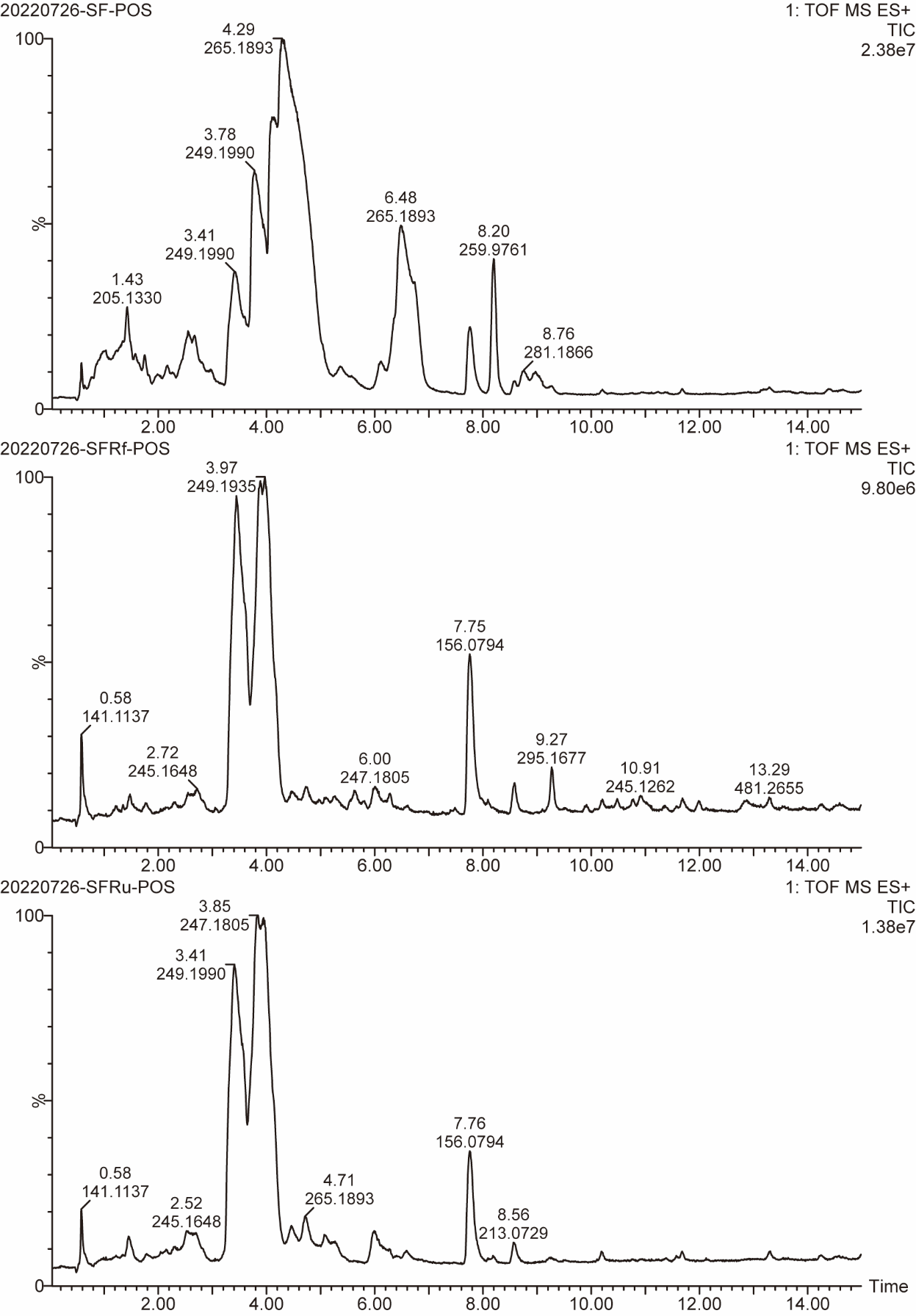


**Figure S13A. Chromatogram of *Sophora flavescens* radix medicinal materials and *Sophora flavescens* radix residue.** SF: *Sophora flavescens* radix medicinal materials; SFRf: fermented *Sophora flavescens* radix residues; SFRu: unfermented *Sophora flavescens* radix residues.


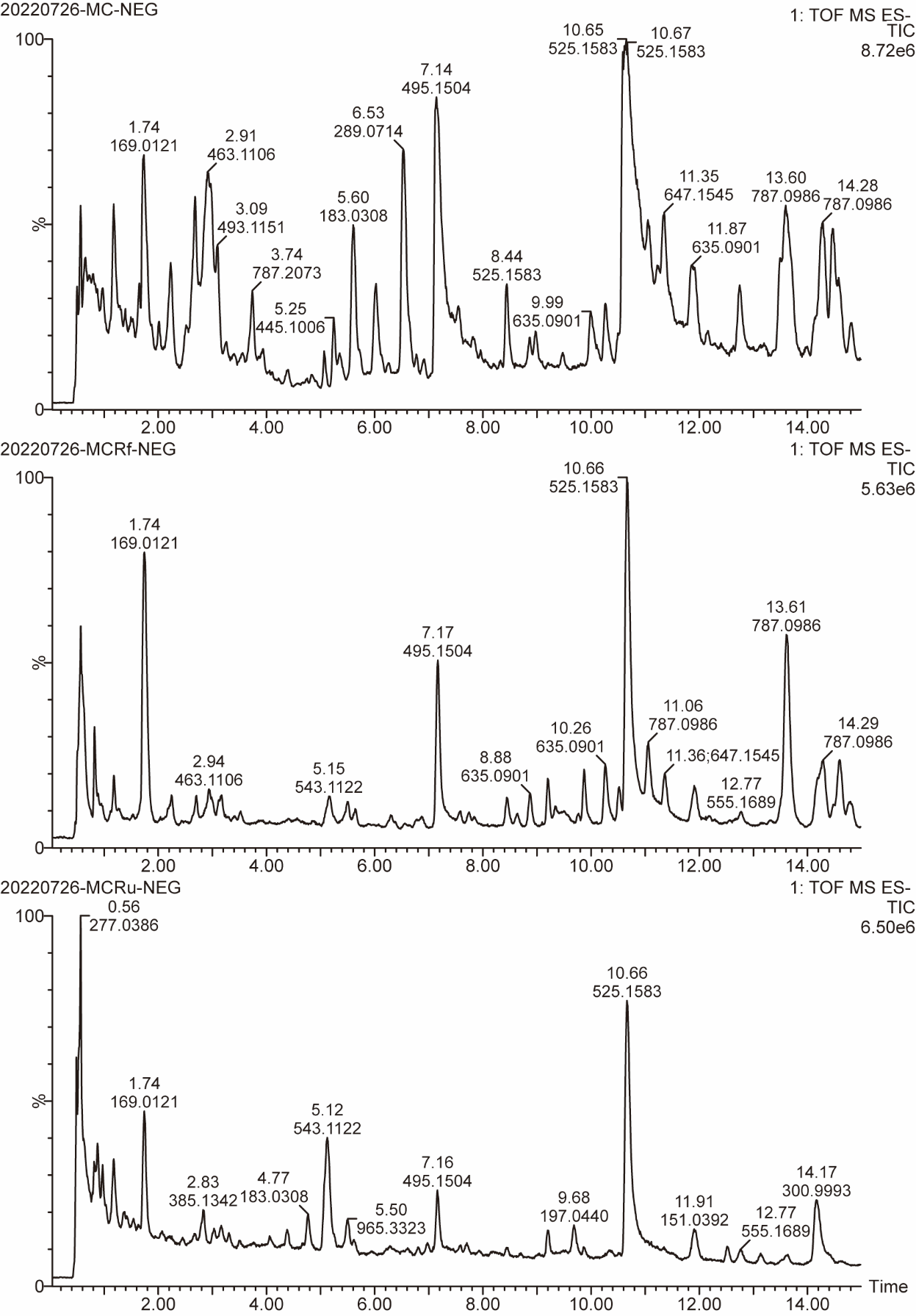


**Figure S13B. Chromatogram of *Moutan cortex* medicinal materials and *Moutan cortex* residue.** MC: *Moutan cortex* medicinal materials; MCRf: fermented *Moutan cortex* residue; MCRu: unfermented *Moutan cortex* residue.
